# Ambient methane functionalization initiated by electrochemical oxidation of a vanadium (V)-oxo dimer

**DOI:** 10.1038/s41467-020-17494-w

**Published:** 2020-07-23

**Authors:** Jiao Deng, Sheng-Chih Lin, Jack Fuller, Jesus A. Iñiguez, Danlei Xiang, Di Yang, Gary Chan, Hao Ming Chen, Anastassia N. Alexandrova, Chong Liu

**Affiliations:** 1 Department of Chemistry and Biochemistry, University of California, Los Angeles, Los Angeles, CA 90095 USA; 2 Department of Chemistry, National Taiwan University, Taipei, 10617 Taiwan; 3 California NanoSystems Institute, Los Angeles, CA 90095 USA

**Keywords:** Electrocatalysis, Electrocatalysis, Inorganic chemistry

## Abstract

The abundant yet widely distributed methane resources require efficient conversion of methane into liquid chemicals, whereas an ambient selective process with minimal infrastructure support remains to be demonstrated. Here we report selective electrochemical oxidation of CH_4_ to methyl bisulfate (CH_3_OSO_3_H) at ambient pressure and room temperature with a molecular catalyst of vanadium (V)-oxo dimer. This water-tolerant, earth-abundant catalyst possesses a low activation energy (10.8 kcal mol^‒1^) and a high turnover frequency (483 and 1336 hr^−1^ at 1-bar and 3-bar pure CH_4_, respectively). The catalytic system electrochemically converts natural gas mixture into liquid products under ambient conditions over 240 h with a Faradaic efficiency of 90% and turnover numbers exceeding 100,000. This tentatively proposed mechanism is applicable to other d^0^ early transition metal species and represents a new scalable approach that helps mitigate the flaring or direct emission of natural gas at remote locations.

## Abstract (web summary)

The undesirable geological release of methane at remote locations can be lessened through an efficient methane conversion process. Here, the authors report selective ambient functionalization of methane by a vanadium (V)-oxo electrocatalyst with a low activation energy and a high turnover frequency.

## Introduction

**Fig. 1:**
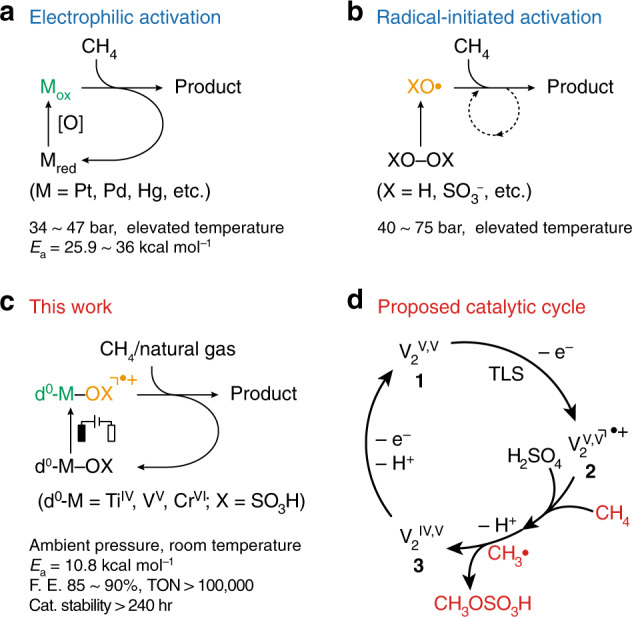
Pathways to CH_4_ functionalization. **a**–**c** Representative approaches of CH_4_ functionalization based on electrophilic activation^3–6^ (**a**) and radical chain mechanism^8–10^ (**b**), in comparison with the proposed electrocatalytic method (**c**). **d** The proposed catalytic cycle of electrocatalytic CH_4_ activation with d^0^ vanadium (V)-oxo dimer (**1**). M_red_ and M_ox_, reduced and oxidized metal active sites, respectively; [O], chemical or electrochemical oxidation; *E*_*a*_, activation energy; d^0^–M, d^0^ early transition metal species; F.E. Faradaic efficiency, TON turnover number, TLS turnover-limiting step.

The wide geological distribution of natural gas resources leads to an undesirable loss of methane (CH_4_) especially at remote locations via flaring or direct emission into the atmosphere^1,2^. One possible strategy to mitigate such an issue is to convert CH_4_ into liquid chemicals at the source of emission under ambient condition with minimal reliance on an industrial infrastructure^2^. Fundamentally, this catalytic conversion requires low activation energy and high reactivity, in order to accommodate the low thermal energy and partial pressure of CH_4_ substrate at ambient condition. Existing approaches of CH_4_ functionalization usually operated at high pressure and/or elevated temperature^3–16^, involve metal-catalyzed reactions^3–7^, superacid-based activation^17^, or catalysis based on peroxo species for a free-radical chain mechanism^8–10^. For the metal-catalyzed reactions reported by Periana and others (Fig. 1a)^3–6^, electrophilic activation of CH_4_ is followed by an oxidation process that regenerates the metal active sites, mostly precious metals including Pt and Pd. Yet, in ambient conditions, the reactivities of these electrophilic metal species seem insufficient to activate CH_4_. In the approaches based on radical chain mechanism (Fig. 1b)^8–10^, initiators including peroxo species yield oxygen radicals that activate CH_4_ at room temperature with low activation energies^18^. But it is uneasy to achieve a sustained, selective catalytic process that generates and replenishes radical species. The challenge of balancing the reactivity and regeneration of active species call for an alternative approach for ambient CH_4_ functionalization.

We propose that a controlled electrochemical generation of oxygen radicals is capable to address the above-mentioned challenge, as electrochemical redox process provides a sustained method of replenishing radical species at ambient conditions without sacrificing their high reactivities^19,20^. While d^0^ early transition metal centers are not known to be directly oxidizable, we accidentally found that electrochemical oxidation of d^0^ early transition metal-oxo species in 68–98% H_2_SO_4_ yields cation radicals on the sulfonic ligand that selectively activate CH_4_ at ambient pressure and room temperature (Fig. 1c). Here, we report d^0^ vanadium (V)-oxo dimer (V_2_^V,V^, **1**)^21^ as the model catalyst for mechanistic understanding (Fig. 1d). As the tentative turnover-limiting step (TLS) uncommon for homogenous electrocatalysis, an electrochemical one-electron removal of **1** yields a cation radical (V_2_^V,V •+^, **2**) reactive towards CH_4_, while the catalytic cycle is fulfilled by additional electrochemical oxidation and cation radical regeneration.

## Results

### Discovery of ambient CH_4_ activation with vanadium (V)-oxo catalyst

**Fig. 2:**
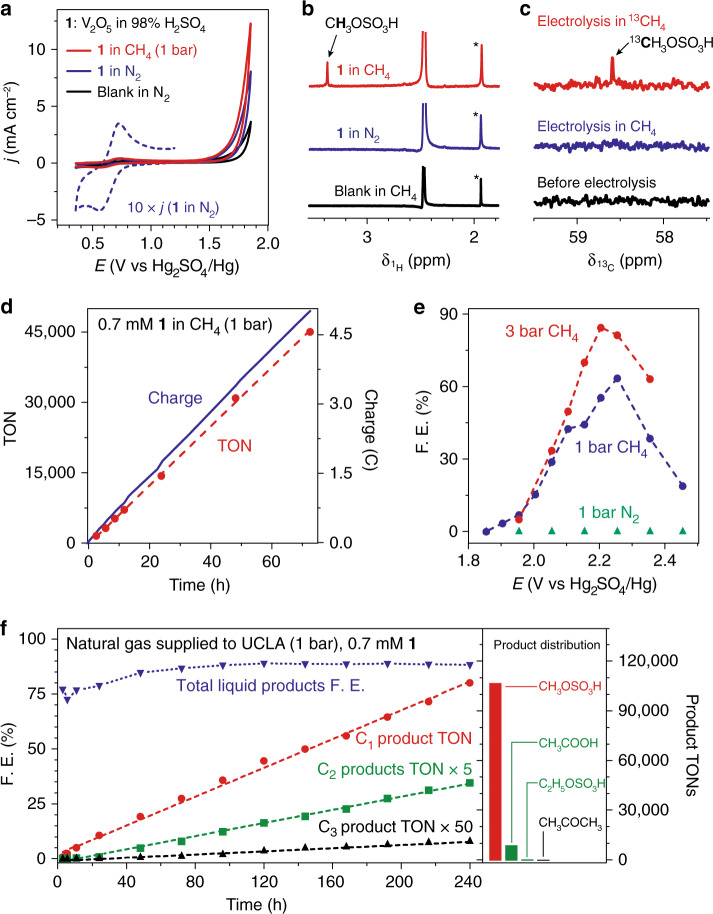
Electrochemical functionalization of CH_4_ and natural gas mixture. **a**, **b** Cyclic voltammograms (**a**) and ^1^H NMR spectra of liquid samples after 6-h electrolysis (**b**) for 10 mM **1** in 1-bar CH_4_ (red), 10 mM **1** in 1-bar N_2_ (blue), and blanks without **1** (black). Dashed blue, current density (*j*) of blue trace magnified by a factor of 10. *, internal standard acetic acid. **c** ^13^C NMR spectra of samples before (black) and after electrolysis with ^13^CH_4_ (red) and CH_4_ of natural abundance (blue), respectively. **d** Calculated TONs (red) and electric charges passed (blue) versus the duration of electrolysis. **e** F.E. of CH_4_ functionalization in 10 mM **1** vs. electrode potential *E* under 1-bar N_2_ (green), 1-bar CH_4_ (blue) and 3-bar CH_4_ (red). **f** Cumulative TONs for C_1_ (red), C_2_ (green, multiplied by a factor of 5), and C_3_ products (black, multiplied by a factor of 50) as well as F.E. values of total liquid products (blue) are plotted against the duration of bulk electrolysis. TON values for CH_3_OSO_3_H (red), CH_3_COOH plus C_2_H_5_OSO_3_H (green), and CH_3_COCH_3_ (black) within 240 h are shown on the right, respectively. Natural gas mixture supplied by SoCalGas was used as the substrate at ambient pressure. Trace products beyond C_3_ were also observed. 100 mV s^−1^ and Pt working electrode in (**a**); FTO working electrode in (**b**) to (**f**), and *E* = 2.255 V vs. Hg_2_SO_4_/Hg in (**b**) to (**d**), and (**f**).

The d^0^ vanadium (V)-oxo catalyst (**1**) was prepared by dissolving V_2_O_5_ in 98% H_2_SO_4_. Cyclic voltammograms of 10 mM **1** in 98% H_2_SO_4_ under 1-bar nitrogen gas (N_2_) (blue), 1-bar CH_4_ (red), and a blank control (black) are displayed in Fig. 2a at 25 ºC on a platinum (Pt) working electrode. A quasi-reversible peak corresponding to V^V^/V^IV^ redox couple was observed with a midpoint potential *E*_1/2_ = 0.644 V vs. Hg_2_SO_4_/Hg reference electrode, with a diffusion coefficient *D* = 2.18 × 10^−11^ m^2^ s^−^^1^ for **1** based on Randles–Savcik analysis (Supplementary Fig. 1a, b)^22^. Additional oxidation current of **1** was observed at the electrochemical potential *E* > 1.6 V vs. Hg_2_SO_4_/Hg, and such an oxidation current is larger in CH_4_ than in N_2_. This observation suggests that **1** can be further oxidized electrochemically and CH_4_ is likely to react with the resultant oxidized species. Bulk electrolysis in 98% H_2_SO_4_ under 1-bar CH_4_ was conducted at a *E* = 2.255 V vs. Hg_2_SO_4_/Hg for 6 h with an electrode of fluorine-doped tin oxide (Supplementary Fig. 1c). The liquid composition after electrolysis were analyzed by ^1^H and ^13^C nuclear magnetic resonance (NMR) spectroscopy. CH_3_OSO_3_H, which yields methanol after hydrolysis^11^, was observed at chemical shift *δ* = 3.34 ppm in ^1^H NMR after electrolysis with 10 mM **1** under CH_4_ (red in Fig. 2b). No gaseous or liquid products other than CH_3_OSO_3_H were observed as a product of CH_4_ oxidation within our detection limit via NMR spectroscopy (Fig. 2b), mass spectroscopy (Supplementary Fig. 2a, b) and gas chromatography (Supplementary Fig. 3). Organic products were not detected in the absence of **1** under CH_4_ (black), with 10 mM **1** under N_2_ (blue in Fig. 2b), or at a less anodic potential (*E* = 1.855 V vs. Hg_2_SO_4_/Hg) (Supplementary Fig. 2d). These data confirm that CH_4_ undergoes a two-electron oxidation into CH_3_OSO_3_H, initiated by the electrochemical oxidation of **1**. The absence of a well-defined redox wave preceding the current onset in Fig. 2a suggests either a homogenous electrocatalysis limited by the rate of electron transfer or the occurrence of materials deposition during the scans of cyclic voltammetry. The measurement of X-ray photoelectron spectra on a FTO electrode after 6-h electrolysis with 10 mM **1** detected no residual V signal on the electrode (Supplementary Fig. 4) despite the observed CH_3_OSO_3_H formation (1-bar CH_4_, *E* = 2.255 V vs. Hg_2_SO_4_/Hg). Reusing this vanadium-exposed FTO electrode for 6-h electrolysis in neat 98% H_2_SO_4_ under CH_4_ yielded no CH_4_ activation. These results suggest a likely homogenous electrocatalysis limited by charge transfer. We also conducted isotope-labeling experiments by introducing ^13^CH_4_ as the substrate at 1-bar pressure. The introduction of ^13^CH_4_ in lieu of CH_4_ of natural abundance leads to the surge of ^13^CH_3_OSO_3_H signal at *δ* = 58.6 ppm in ^13^C NMR after electrolysis (Fig. 2c). An introduction of a 50% ^13^C-enriched CH_4_ yields not only the same peak in ^13^C NMR but also a triplet in ^1^H NMR with ^13^C–^1^H doublet and ^12^C–^1^H singlet at a 1:1 ratio of integration area (Supplementary Fig. 5). The optical absorption spectra of the solution before and after electrolysis were identical to each other (Supplementary Fig. 2e), implying that **1** as a catalyst was regenerated after electrolysis.

### Electrochemical CH_4_ and natural gas functionalization

The electrocatalysis with **1** is durable and selective for functionalization of CH_4_ with high turnover numbers (TONs) and turnover frequencies (TOFs). Bulk electrolysis was conducted with 0.7 mM **1** at 25 ºC under 1-bar pressure of CH_4_. Liquid aliquots at different time points were analyzed, and the electrochemical TONs were calculated based on the existing method^19,23^. Figure 2d displayed the amount of electric charge and the calculated TONs as a function of electrolysis duration. A linear correlation suggests a durable catalyst of TON up to 45,000 in 72 h without signs of catalyst degradation. We also investigated the Faradaic efficiency (F.E.), defined as the selectivity of converting CH_4_ into CH_3_OSO_3_H based on the amount of electric charge, as function of *E* at 25 ºC. In 10 mM **1** (Fig. 2e), the absence of CH_4_ leads to no product formation (green), and under 1-bar CH_4_ an optimal F.E. = 63.5% when *E* = 2.255 V vs. Hg_2_SO_4_/Hg (blue). We found that the reaction selectivity is limited by the mass transport and a limited solubility of CH_4_ in solvent (~1 mM)^11^. When CH_4_ pressure increased to 3 bar, the optimal F.E. = 84.5% at *E* = 2.205 V vs. Hg_2_SO_4_/Hg (red in Fig. 2e). The corresponding TOFs of **1** as an electrocatalyst are 483 and 1336 h^−1^ for CH_4_ at 1 and 3-bar pressures, respectively, which are conservative and underestimated given the nature of our analysis (Supplementary Note 1). The measured TOF values at room temperature compare quite favorably with other reported catalysts at elevated temperatures and high pressures (Supplementary Table 1).

We expanded the substrate scope from CH_4_ to ethane (C_2_H_6_), propane (C_3_H_8_), and ultimately natural gas mixture at 1-bar pressure. C_2_H_6_ was oxidized to a mixture of acetic acid (CH_3_COOH) and ethyl bisulfate (C_2_H_5_OSO_3_H) (Supplementary Fig. 6a), whose TOF values are 297 and 235 h^−1^, respectively. C_3_H_8_ was converted to mostly isopropyl bisulfate (*i-*C_3_H_7_OSO_3_H) with trace acetone (CH_3_COCH_3_) in a 6-h electrolysis (Supplementary Fig. 6b), with TOF values of 962 and 2 h^−1^, respectively. One challenge in designing a process of natural gas utilization is to balance the low reactivity of the major component CH_4_ with the high reactivity of minor light alkane components^24^, as in some cases the latter substrates can react 100-time faster than CH_4_^25^. The similar TOF values among CH_4_, C_2_H_6_, and C_3_H_8_ reported here renders **1** a suitable candidate of direct natural gas utilization at ambient conditions without much upstream separation.

Natural gas supplied to UCLA via pipeline by SoCalGas^26^ was used as the substrate of electrolysis (*E* = 2.255 V vs. Hg_2_SO_4_/Hg) with 0.7 mM **1** in 98% H_2_SO_4_ at room temperature and ambient pressure. Powered by electricity, natural gas was oxidized into organic chemicals while dioxygen in air was reduced on the counter electrode, fulfilling a partial oxidation of natural gas with air with a net reaction of CH_4_ + 1/2O_2_ + H_2_SO_4_ → CH_3_OSO_3_H + H_2_O. The yielded products mainly consist of CH_3_OSO_3_H from CH_4_, CH_3_COOH, and C_2_H_5_OSO_3_H from C_2_H_6_, and CH_3_COCH_3_ from C_3_H_8_ in the natural gas. The TONs of C_1_–C_3_ products reached about 107,000, 9300, and 200 within 240 h, respectively (Fig. 2f) with the final concentration of CH_3_OSO_3_H approaching 10 mM (Supplementary Fig. 7). The observed one-order-of-magnitude difference between **1** and CH_3_OSO_3_H product again supports the catalytic feature of our observation. The total F.E. of all liquid products remain stable at around 90% during electrolysis after an initial induction period (Fig. 2f, Supplementary Table 2). The linear relationship between TONs and electrolysis durations suggest that **1** remains active and is tolerant to the impurities in natural gas mixture. Previous analysis suggests that a lower H_2_SO_4_ concentration in the electrolyte, ideally below 80%, is needed for industrial implementation^11^. This requirement is against the thermodynamic limit of reactions in H_2_SO_4_ with SO_3_ as the oxidant^11^. Yet we found that **1** remains active towards CH_4_ functionalization in aqueous solution with H_2_SO_4_ concentrations as low as 68% under 25 ºC and 1-bar CH_4_ (Supplementary Fig. 6c), yielding a mixture of methanol and CH_3_OSO_3_H (Supplementary Fig. 6d). The robustness under prolonged operation and the applicability in diluted H_2_SO_4_-H_2_O mixed solvent renders the catalyst potentially suitable to be employed to functionalize natural gas with minimal maintenance.

### Scale-up potential over vanadium (V)-oxo electrocatalyst

The reported catalyst **1** is also capable to yield high product concentrations amenable for practical implementations. Due to the limited solubility of CH_4_ in the solvent within current batch reactor^11^, electrocatalytic experiments of higher product concentrations were conducted under room temperature at 11-bar CH_4_ pressure, in order to mitigate the mass transport issue of limited gas solubility (vide supra) (Supplementary Fig. 7). A 72-h electrolysis leads to a CH_3_OSO_3_H concentration of ~110 mM with F.E. = 81.2% (*E* = 2.376 V vs. Hg_2_SO_4_/Hg). Adding 1 M CH_3_OSO_3_H prior to the electrocatalysis under the same condition does not hinder the catalysis or decompose the pre-added CH_3_OSO_3_H. A single-pass conversion of 1% was observed in the mixed-flow electrochemical reactor, comparable to the results that lead to electrochemical reduction of CO_2_ and CO at near-industrial level^27,28^. The product concentrations reported here are higher than the ones in other electrocatalysis^5,6^, and suggest that high product concentration of electrocatalysis is attenable. There seems no observable fundamental limit for product concentrations exceeding one mole per liter, a threshold considered suitable for industrial applications^11^.

### Kinetics investigation of electrochemical CH_4_ functionalization

**Fig. 3:**
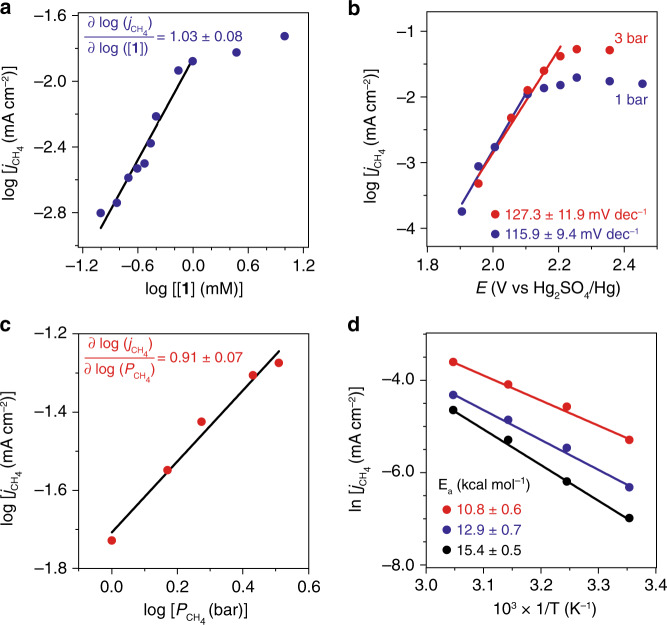
Electrocatalytic kinetics. **a** The logarithmic of partial current density for CH_4_ functionalization, log(*j*_CH4_), versus the logarithmic of **1**’s concentration, log([**1**]). **b** Log(*j*_CH4_) vs. *E* under CH_4_ pressures of 1 bar (blue) and 3 bar (red) with the fitted Tafel slopes displayed. **c** Log(*j*_CH4_) vs. the logarithmic of CH_4_ pressure, log(*p*_CH4_). **d** The natural logarithmic of partial current density for CH_4_ functionalization, ln(*j*_CH4_), vs. inverse of temperature, T^−1^, at 1.955 V (black), 2.005 V (blue), and 2.055 V (red) vs. Hg_2_SO_4_/Hg, respectively. The corresponding apparent activation energies (*E*_*a*_) are displayed. Unless noted specifically, 25 °C, 10 mM **1** in 98% H_2_SO_4_, *E* = 2.255 V vs. Hg_2_SO_4_/Hg, *p*_CH4_ = 1 bar, data recorded from 6-h bulk electrolysis.

The attractive feature of catalyst **1** led us to investigate the underlying mechanism during electrolysis with CH_4_ as the substrate. The current density corresponding CH_3_OSO_3_H formation (*j*_CH4_), a surrogate of CH_4_-activating rate, was investigated as a function of catalyst concentration [**1**] (Fig. 3a), the electrode potential *E* (Fig. 3b), the partial pressure of CH_4_ (*p*_CH4_) (Fig. 3c), and the temperature T (Fig. 3d). A linear relationship with a slope = 1.03 ± 0.08 between log_10_(*j*_CH4_) and log_10_([**1**]) suggests that CH_4_ activation is first-order on **1** (Fig. 3a). When log_10_(*j*_CH4_) was plotted against *E* (Fig. 3b), a Tafel slope of about 120 mV dec^−1^ was observed before *j*_CH4_ plateaus at larger *E* values as CH_4_ is depleted near electrode. This suggests that the first electron removal from **1** is the TLS, uncommon for homogeneous electrocatalysis (Supplementary Note 2). The overlapping points under 1 and 3-bar CH_4_ pressure when *E* < 2.1 V vs. Hg_2_SO_4_/Hg suggest that CH_4_ is not involved in the TLS or any pre-equilibrium steps. When *E* > 2.1 V vs. Hg_2_SO_4_/Hg, a linear relationship between log_10_(*j*_CH4_) and log_10_(*p*_CH4_) with a slope of 0.91 ± 0.07 (Fig. 3c) suggests that CH_4_ is activated in a first order after the TLS. We also found that the residual current density, the difference between total current density (*j*_total_) and *j*_CH4_, is independent of *p*_CH4_ (Supplementary Fig. 2c). It further corroborates that no gaseous or liquid products other than CH_3_OSO_3_H were generated from CH_4_ oxidation, and solvent oxidation into O_2_ and possibly trace persulfate^29,30^ is the only plausible side reaction (vide infra). The Arrhenius plot between ln(*j*_CH4_) and 1/T yields an apparent activation energy (*E*_*a*_) as low as 10.8 ± 0.6 kcal mol^−1^ (Fig. 3d), consistent with the observed ambient reactivity. When conducting electrolysis of **1** in 98% D_2_SO_4_ with CH_4_ of natural isotope abundance, ^2^D and ^1^H NMR spectra showed no H/D exchange in the methyl group of product CH_3_OSO_3_H (Supplementary Fig. 6e). This excludes the possible mechanism induced by an electrochemically generated superacid^17^, which should yield significant *H*/*D* exchange in the methyl group^11^. In addition, when CH_4_ was exposed to **1** in 98% H_2_SO_4_ with added K_2_S_2_O_8_ or H_2_O_2_ in the absence of electricity, CH_4_ functionalization was not observed at ambient conditions (Supplementary Fig. 6f). This illustrates that it is difficult for chemical method to sustainably generate reactive radical intermediates at room temperature, which necessitates our use of electrochemistry as proposed before. It also shows that the possible formation of peroxo species including peroxoacids is not contributing to the observed reactivity. When the electrolyte was switched from 98% H_2_SO_4_ to oleum with 20% SO_3_, a 5.4:1 molar ratio between CH_3_SO_3_H and CH_3_OSO_3_H was observed after electrocatalysis (Supplementary Fig. 8). This indicates the formation of CH_3_• radical during the catalysis, which yields CH_3_SO_3_H in the presence of SO_3_^9^. Overall, our experimental data support an electrochemical catalysis of low activation energy. After a turnover-limiting one-electron oxidation of **1**, the oxidized species undergoes a first-order C–H activation in CH_4_ and a formation of CH_3_• radical (Fig. 1d).

### Unveiling dimer structure of vanadium (V)-oxo catalyst

**Fig. 4:**
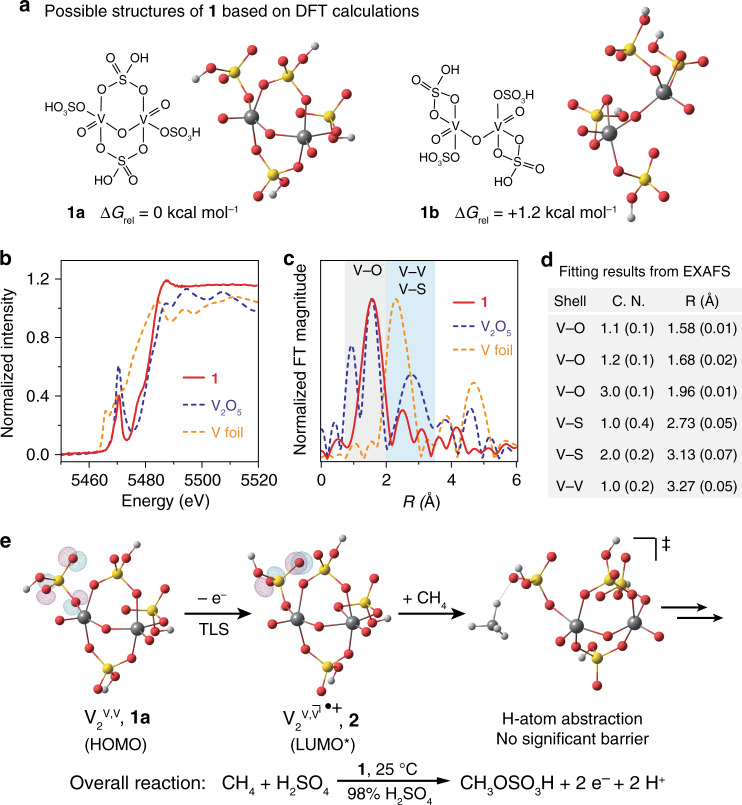
Structural information of the catalyst and a proposed mechanism. **a** Possible isomeric structures of **1** and their relative energetics based on DFT calculations. **b**, **c** Normalized intensity of V K-edge X-ray absorption near-edge structure (XANES) (**b**) and extended X-ray absorption fine structure (EXAFS) (**c**) for **1** (solid red), V_2_O_5_ (dashed blue), and metallic V (dashed yellow). **d** Calculated coordination number (C.N.) and the distance (*R*) away from V atom based on EXAFS results. **e** Calculated frontier orbitals involved in the TLS and the proposed transition state of C–H activation step. HOMO highest occupied molecular orbital, LUMO lowest unoccupied molecular orbital. *Designated when considering spin–orbital coupling, equivalent to singly occupied molecular orbital (SOMO) in restricted formalism.

Despite its ease of preparation, the structural information of **1** is not well understood. It was hypothesized to be a V_2_^V,V^ dimer with two terminal V^V^≡O moieties connected by a bridging oxo^21^. We measured **1**’s optical absorption (Supplementary Fig. 9a) and the ^51^V NMR spectrum (Supplementary Fig. 9b), which confirmed that **1** is different from monometallic VO_2_^+^ species in an aqueous medium Density functional theory (DFT) calculations suggest that **1** may exist as two isomers, **1a** and **1b** (Fig. 4a), with a calculated energy difference of 1.2 kcal mol^−1^. In an attempt to obtain the correct structure of **1** and real conformation in solution, X-ray absorption spectroscopy of V atom was conducted for 10 mM **1** in 98% H_2_SO_4_, solid V_2_O_5_, and metallic V foil (solid red, dashed blue, and dashed yellow in Fig. 4b, c, respectively). We carried out a least-square-regression analysis on X-ray absorption near-edge structure (XANES) for the threshold positions, the first peak in the derivative spectra, of VO, V_2_O_3_, VO_2_, and V_2_O_5_ to determine the electronic structure and oxidation state of vanadium in **1** (Supplementary Fig. 9c)^31^. The electronic structure of vanadium in **1** remains similar to that of vanadium in V_2_O_5_, confirming the d^0^ electronic structure of vanadium. The extended X-ray absorption fine structure (EXAFS) can offer coordination information of absorbing atoms by extracting the structural parameters. As shown in Fig. 4c, the absence of noticeable peaks in the region beyond 4 Å (solid red), compared with those of V_2_O_5_ and V foil (dashed blue and dashed yellow, respectively), indicates that **1** is a complex homogenously dispersed in the solvent. The peak around 1.56 Å in **1**’s EXAFS spectrum (gray area) is attributed to the V–O bonds, following the assignment of V–O bonds in the V_2_O_5_ sample. While this comparison provides some information, the general low symmetries of the vanadium-based species prevent us from gaining detailed structural information of **1** solely based on EXAFS data^32^. To this end, we conducted the fitting of **1**’s V K-edge EXAFS spectrum combining the structure suggested by DFT calculations (shown in Fig. 4d). It reveals that the central V atoms are penta-coordinated by O atoms with three types of V–O bond lengths (1.58, 1.68, and 1.96 Å) in the first coordinated shell, with a bridging oxo with a V–O bond length of 1.68 Å. We note that the EXAFS of **1** suggests a unique coordination environment 2.0–3.5 Å away from V atom (blue area), which is different from the monometallic VO_2_^+^ species in aqueous medium (Supplementary Fig. 9d). The fitting results of second shell (blue area) indicate that consistent with the predicted structure **1a**, there are not only three S atoms in the second shell (2.73 and 3.13 Å) but also one V atom at the distance of 3.27 Å away from the central V atom (Supplementary Table 3). Detailed analysis is provided in the Supplementary information (Supplementary Fig. 9e, f). These results reveal the existence of a hypothesized structure of µ-oxo bridged V_2_^V,V^ dimer^33^, and suggest that **1a** is the structure of **1** in the solution.

### Operando characterizations for mechanistic study

Additional operando characterizations were conducted to confirm a homogenous electrocatalysis and elucidate identities of immediate species. Operando Raman spectroscopy measures the vibrational spectra of chemical species near the FTO electrode at different values of *E* in CH_4_. No spectral changes were observed and the vibrational spectra evidently differs from solid V_2_O_5_ sample (Supplementary Fig. 10). This suggests that there is no detectable heterogeneous intermediate deposited on the FTO electrode during electrocatalysis. Operando XAS spectra of V atom was also measured at different values of *E* in CH_4_. Largely, the XANES and EXAFS spectra (Supplementary Figs. 11 and 12) differ from the ones of solid V_2_O_5_ sample and supports a homogenous electrocatalysis. Yet some information of reaction intermediates is available. The formal oxidation state of V species, indicative in the pre-edge region of XANES spectra (Supplementary Fig. 11), decreases from +5 to near +4 with increasing value of *E*, contrary to the typical trend observed in heterogeneous catalysts of electrochemical oxidation^34^. This reveals the presence of mixed-valence V_2_^IV,V^ during catalysis (Supplementary Note 3). It also supports a homogenous, diffusion-controlled catalysis, since a hypothetical immobile V(IV) species deposited on the electrode may not have long enough lifetime to be detectable (Supplementary Note 3), given the large thermodynamic driving force of oxidizing V(IV) (>1.2 V from Fig. 2a). The pre-edge region also witnesses a broadening and intensity decrease of the pre-edge peak concurrent with the increase of *E* and the observation of electrocatalytic CH_4_ functionalization (Supplementary Fig. 11). This suggests an increase of coordination symmetry near V atom and possibly a loss of sulfonic ligand^31^. The operando EXAFS results (Supplementary Fig. 12) also displays a decrease of average coordination number of sulfonic ligands per V atom concurrent with increasing *E* values. Those results imply that the bisulfate group in CH_3_OSO_3_H likely originate from the vanadium catalyst, a plausible radical rebound mechanism^35^.

### Proposed electrocatalytic cycle with vanadium (V)-oxo dimer

Combining experimental and computational results, we established a proposed catalytic cycle of **1** for CH_4_ functionalization (Fig. 1d) despite its uncommon assignment of TLS that warrants additional investigation (Supplementary Note 2). A turnover-limiting electrochemical oxidation of **1a** removes one electron from O 2p orbitals in the sulfonic ligand, which is calculated as the highest occupied molecular orbital (HOMO) of **1a** (Fig. 4e). The resultant cation radical **2** is predicted to possess an empty frontier spin-orbital on the same O 2p orbitals (lowest unoccupied molecular orbital (LUMO) of **2** in Fig. 4e), which is postulated to initiate H-atom abstraction from CH_4_. DFT calculations predict reaction trajectory between **2** and CH_4_ without significant energy barrier (Fig. 4e, Supplementary Fig. 13a). This is consistent with our experimental observation that the TLS is the one-electron oxidation of **1** other than the step of C–H activation (Supplementary Note 2). The calculated barrier-less C–H activation also helps explain the similar TOFs toward various light alkanes in the natural gas^24^. Currently, we were unable to experimentally characterize **2** and the H-atom abstraction step due to its transient nature, which will be of our research focus in the future. Yet the subsequent steps of CH_4_ functionalization seems to proceed with the formation of CH_3_• and a radical rebound process^35^. This leads to a two-electron oxidation and CH_3_OSO_3_H formation with a ligand loss on a V_2_^IV,V^ dimer (**3**), which will be readily re-oxidized electrochemically to regenerate **1** (Fig. 1d, Supplementary Fig. 14).

### Exploration of molecular and material variants for vanadium (V)-oxo catalyst

DFT calculations of the atomic charges^36^ suggest that cation radical **2** is stabilized thanks to orbital delocalization, in comparison to the scenario when one electron was removed from sulfuric acid (Supplementary Fig. 13b, c). This is consistent with the results that the calculated ionization energies of **1a** is lower by 12–14 kcal mol^−1^ than that of sulfuric acid. This implies that the metal-oxo centers as carrier of sulfonic ligands stabilize the electrochemically generated cation radical, at the same time maintain a cation radical reactive enough for ambient CH_4_ functionalization. Other d^0^ early transition-metal-oxo species can possess similar reactivities. We found that d^0^ metal-oxo species, including Ti^IV^-oxo and Cr^VI^-oxo, are also electrochemically active towards functionalizing CH_4_ at ambient conditions (Supplementary Fig. 15). A more extensive survey over the first half of the Period 4 elements except Sc indicates that only Ti, V, Cr, and possibly Mn display similar reactivities (Supplementary Fig. 16). It suggests the strategy of employing d^0^ early transition-metal-oxo species is generally applicable for ambient electrochemical functionalization of natural gas. As such a general trend of reactivity was not observed before, we posit our electrochemical approach may offer new perspective towards the challenge of CH_4_ functionalization.

Practically, a heterogeneous catalyst variant may also be desirable. While **1** is characterized as a homogenous catalyst, we found a two-dimensional layered material, VOPO_4_∙2H_2_O (**4**) with exposed V-oxo edges^37^ (Supplementary Fig. 17a, b), acts as a heterogeneous variant of **1** in 98% H_2_SO_4_ (Supplementary Fig. 17c, d). This preliminary result suggests that even within the same metal-oxo system, the catalyst subsequently its reactivity can be tuned with additional materials design and engineering. This heterogenous variant also simplifes product separation in downstream process, thanks to the absence of vanadium in the liquid phase.

## Discussion

Overall, the general tunability of catalyst composition may herald better catalysts with higher TOF, lower oxidation potential, as well as versatile design of the overall process. The ambient condition of reported catalysis facilitates the use of O_2_ in ambient air as the terminal electron acceptor, as well as the use of ambient natural gas feedstock for onsite functionalization without a centralized facility. Future research will focus on possible scale-up with the exploration of optimal reaction conditions. The employment of 98% H_2_SO_4_ or more diluted acids, other than oleum, mitigates the generation of excessive acid in product separation and is attractive for practical application^38^. Additional fundamental and engineering investigation, including the employment of gas diffusion electrode^39^ as well as ingenious design of electrochemical reactors^28^, will further explore the possible application of converting CH_4_ into commodity chemicals with minimal infrastructure support at remote locations. This will lead to the more efficient usage of green-house gases and reducing their emission into atmosphere.

## Methods

### Chemicals and materials

All chemicals were purchased from Sigma-Aldrich, Thermo Fisher Chemical, or VWR International, unless otherwise stated. All chemicals were used as received unless specified. Dimethyl sulfoxide-d_6_ (DMSO-d_6_) was purchased from Cambridge Isotope Laboratories, Inc. All deionized (DI) water was obtained from a Millipore Milli-Q Water Purification System. Fluorine-doped Tin Oxide (FTO) glass was purchased from Hartford Glass Incorporation. CH_4_ (99.5%) was purchased from Airgas, C_2_H_6_ (99%), C_3_H_8_ (98%), and ^13^CH_4_ (99%; 99 atom% ^13^C) were purchased from the Sigma-Aldrich. Natural gas mixture was obtained from the outlet in Molecular Science Building 4211, Department of Chemistry and Biochemistry, UCLA, which is supplied via pipeline by SoCalGas. SRI multiple gas analyzer #5 gas chromatograph (GC), 8610C is used to analyze the natural gas mixture. The components are 91.78 mole% CH_4_, 4.31 mole% C_2_H_6_, 0.31 mole% C_3_H_8_, 0.04 mole% *n*-C_4_H_10_, 0.03 mole% *i*-C_4_H_10_, 0.01 mole% *n*-C_5_H_12_, 0.01 mole% *i*-C_5_H_12_, and 0.81 mole% CO_2_. Unless specifically noted, reagent-grade 98% H_2_SO_4_ (VWR BDH3074-3.8LP) was employed as the solvent, which contains 5 ppm of metal impurities. When needed, we also employ high-purity 98% H_2_SO_4_ (Sigma-Aldrich 339741), which contains 0.3 ppm of metal impurities as shown in the product certificate.

### Chemical and material characterizations

Ultraviolet–visible (UV–vis) spectra was conducted on Hewlett-Packard 8453 UV–vis spectrophotometer. Proton NMR (^1^H-NMR) and carbon NMR (^13^C-NMR) were recorded on a Bruker AV400 (400 MHz) spectrometer. Deuterium NMR (^2^D-NMR) was recorded on a Bruker AV500 (500 MHz) spectrometer. Vanadium NMR (^51^V-NMR) was performed on an Agilent DD2 600 (600 MHz) spectrometer. Powder X-ray diffraction (XRD) patterns were measured on a Panalytical X’Pert Pro X-ray Powder Diffractometer with a Cu Kα source (*λ* = 1.54178 Å), The intensities were recorded within the 2*θ* range from 10° to 60° with a voltage of 45 kV, and a current of 40 mA. Scanning electron microscope image was measured with a JEOL JSM 6700F instrument. XANES and EXAFS were recorded at BL17C of National Synchrotron Radiation Research Center (NSRRC), Hsinchu, Taiwan. Gas chromatography–mass spectrometry (GC-MS) was performed on Agilent 6890-5975 GC-MS with Inert XL Selective Detector. The GC is equipped with a capillary HP-5MS column (Model No.: 19091S-433, 30.0 m × 250 μm × 0.25 μm). The instrument is operated with an oven temperature of 50 ºC, an inlet temperature of 280 ºC, and a flow rate of 1.2 mL min^−1^ with helium carrier gas. A split/splitless injector is applied with a split ratio of 5:1 and a split flow of 5 mL min^‒1^. The MS has a source temperature of 230 ºC and a quadrupole temperature of 150 ºC. The SRI multiple gas analyzer #5 GC is equipped with 3 S.S. columns including 18′′ Hayesep D, 3′MS 5A and 6′ Hayesep D. The instrument is operated with an oven temperature of 50 ºC, a temperature profile from 50 to 270 ºC, and a flow rate of 40 mL min^−1^ at 15 psi with argon carrier gas. X-ray photoelectron spectroscopy (XPS) was measured on a Kratos AXIS Ultra spectrometer (Kratos Analytical, Manchester, UK).

### Catalyst preparation

Homogeneous bimetallic catalyst **1** was prepared by dissolving vanadium pentoxide (V_2_O_5_) in 98% H_2_SO_4_ with ultra-sound treatment for 6 h. Homogeneous titanium (IV)-oxo and chromium (VI)-oxo catalysts were prepared by dissolved titanyl sulfate (TiOSO_4_) and potassium chromate (K_2_CrO_4_) in 98% H_2_SO_4_, respectively. The heterogeneous variant **4** (VOPO_4_·2H_2_O) was prepared based on previous literature^37,40^. V_2_O_5_ (4.8 g), H_3_PO_4_ (85.5%, 26.6 mL), and H_2_O (115.4 mL) were refluxed at 110 ºC for 16 h. After gently cooling down to room temperature, the yellow precipitate in the mixture was collected by filtration and washed several times with water and acetone. The resulting sample was dried in an oven at 100 ºC for 3 h. When **4** was investigated for its electrochemical response, **4** was loaded onto a FTO electrode via a dip-coating procedure. A dispersion of **4** was prepared at a concentration of 6 mg mL^−1^ in 2-propanol. The yellow dispersion was ultrasonicated for 30 min until the color of the dispersion became faded. Afterwards, sodium carboxymethyl cellulose (CMC) was added into the dispersion (weight ratio of VOPO_4_·2H_2_O: CMC = 80: 5). The mixture was stirred at 600 revolution per minute (rpm) on the heating plate to remove excess 2-propanol and form a homogenous slurry, which was then dip-coated onto FTO at a loading amount of 1.9 mg cm^−2^ for **4**.

### Electrochemical characterization

All electrochemical experiments were recorded using a Gamry Instruments Reference 600+ and Interface 1000 potentiostats. Unless mentioned specifically, a three-electrode setup was applied with a Pt wire pseudo-reference electrode and a Pt counter electrode. The Pt pseudo-reference electrode was calibrated to a Hg_2_SO_4_/Hg (saturated K_2_SO_4_) reference electrode (CH Instrument, Inc.) via the measurement of open-circuit potentials. The relationship is *E*(V vs. Hg_2_SO_4_/Hg) = *E*(V vs. Pt) + 0.755 V. The gas environment of the electrochemical cell was controlled either CH_4_ (Airgas, 99.5%) or N_2_ (Airgas, 99.999%), which were bubbled into the reactor at rates of 7.2 (CH_4_) and 10 (N_2_) standard cubic centimeters per minute (sccm) with the use of a mass flow controllers (Omega Engineering, Inc., FMA5510A). The data were reported after *iR* compensation. Unless noted specifically, the electrolyte is 98% H_2_SO_4_ with a certain vanadium concentration of **1**.

Experiments of cyclic voltammetry were conducted in a single-chamber electrochemical cell with a 2-mm Pt working electrode (CH Instruments, Inc.). Bulk electrolysis with 5 mL homogeneous catalyst solution was typically conducted in a two-chamber electrochemical cell with a Nafion 117 membrane as the separator and a piece of commercial fluorine-doped tin-oxide (FTO) glass as the working electrode. Here, the FTO glass was encapsulated with Teflon tapes so that the exposed electrode is 1 × 1 cm in dimension. The solution was pre-saturated with CH_4_ or N_2_ for 20 min before the commencement of electrolysis. The background signal contribution of FTO glass was subtracted for the recorded data. Liquid aliquots were taken before, during and after the electrolysis for product analysis. Gaseous samples were taken manually from the outgas of the reactor, diluted with pure N_2_ (1:5 ratio) for transportation purpose, and manually injected into the GC–MS. The injected permanent gas was not well separated by the column, but the MS spectra of the eluted gas peak (*t* ~3.3 min) was capable to quantify permanent gases with a detection limit of ~10 ppm. Additional measurements of gas chromatography for the gaseous effluents was conducted by the SRI multiple gas analyzer #5. The experiments at elevated pressure was conducted into a custom-designed setup utilizing a pressure vessel with two gas feedthrough and three electric feedthroughs (Parr Instrument, Series 4600, 1000 mL). In this setup, the gas pressure was controlled between 1 and 11 bar and a constant gas flow of up to 28.7 sccm was maintained during the electrolysis. The temperature of the electrolysis was maintained by an oil bath at a range between 25 and 55 ºC. For experiments using substrates other than CH_4_, the same procedure is followed except the gas flow rate is set at 10 sccm calibrated against N_2_. In the 240-h electrolysis using natural gas mixture as the substrate, the electrolyte was refilled after each aliquot sampling in order to maintain a constant electrolyte volume. Experiment with 50% ^13^C-enriched CH_4_ was conducted by feeding regular CH_4_ and ^13^CH_4_ gases at equal flow rates controlled by two mass flow controller.

We found that typical reagent-grade 98% H_2_SO_4_ possesses nonzero background activity toward CH_4_ at high electrochemical potential (Supplementary Fig. 16). We argue that such background activity is due to the trace metal impurity, possibly V as H_2_SO_4_ is industrially synthesized by a V_2_O_5_ catalyst in the contact process. The reagent-grade 98% H_2_SO_4_ or oleum employed in most studies contains about 5 ppm of metal impurities (VWR BDH3074-3.8LP). When we employed high-purity 98% H_2_SO_4_ (Sigma-Aldrich 339741) that only contained 0.3 ppm of metal impurities as shown in the product certificate, the observed reactivity of neat 98% H_2_SO_4_ vanished (Supplementary Fig. 16).

When the heterogeneous variant **4** was investigated for its electrochemical response, a similar procedure was followed. However, owing to the difference of solution composition, the Pt pseudo-reference electrode has a different relationship with the Hg_2_SO_4_/Hg reference electrode: *E*(V vs. Hg_2_SO_4_/Hg) = *E*(V vs. Pt) + 0.268 V.

### Attempts of using chemical oxidants at ambient conditions

When CH_4_ (7.2 sccm, 1 bar) was bubbled into a 98% H_2_SO_4_ solution with 10 mM **1** and 10 mM K_2_S_2_O_8_ for 6 h at ambient conditions, the formation of methyl bisulfate (CH_3_OSO_3_H) as a possible product of CH_4_ oxidation was not observed (Supplementary Fig. 6f). Similar experiment was conducted with 10 mM H_2_O_2_ in lieu of K_2_S_2_O_8_. The formation of methyl bisulfate (CH_3_OSO_3_H) as a possible product of CH_4_ oxidation was not observed (Supplementary Fig. 6f), either.

### Quantification of liquid products

^1^H-NMR was applied to quantify product accumulation in DMSO-d_6_ using acetic acid (CH_3_COOH) as the internal standard. Totally, 0.4 mL liquid aliquots from electrolysis were mixed with 0.1 mL DMSO-d_6_ prior to the measurements. Chemical shifts are reported on a parts-per-million (ppm) scale. Methyl bisulfate (CH_3_OSO_3_H) exhibits a singlet at 3.34 ppm while the singlet from acetic acid (CH_3_COOH) peak resides at 1.96 ppm. A calibration curve was constructed by determining the relative ratio of integrated area between the NMR peaks of CH_3_OSO_3_H and CH_3_COOH. Product quantification of C_2_H_6_, C_3_H_8_, and natural gas mixture follows the same protocol, except for the quantification of CH_3_COOH as a C_2_ product from C_2_H_6_. The quantification of CH_3_COOH as a C_2_ product was fulfilled by adding a known concentration of CH_3_OSO_3_H as an internal standard in a separate ^1^H-NMR measurement.

### Calculation of FE

The F.E. of bulk electrolysis was calculated based on the following equation:

1 $${\rm{F.E.}} = \frac{{nFC_{\rm{product}}V_{\rm{solution}}}}{{{\rm{Overall}}\,{\rm{charge}}}} \times 100\%,$$

Here, *F* is the Faraday’s constant, *C*_product_ is the concentration of product after bulk electrolysis, *V*_solution_ is the total electrolyte volume, and the overall charge is the total electric charges passed through the working electrode. The variable *n* in the equation is the number of electrons required in order to generate one product molecule by electrochemistry. *n* = 2 for the formation of methyl bisulfate (CH_3_OSO_3_H) from CH_4_. *n* = 2 and 6 for the formation of ethyl bisulfate (C_2_H_5_OSO_3_H) and acetic acid (CH_3_COOH) from C_2_H_6_, respectively. *n* = 2 and 4 for the formation of isopropyl bisulfate (*i*-C_3_H_7_OSO_3_H) and acetone (CH_3_COCH_3_) from C_3_H_8_, respectively.

### Calculation of TOF and TON

In the following we provide the protocols that we calculate the TOFs and TONs for the reported data, based on the methods established in prior literature^19,23^.

The diffusion coefficient for **1** (*D*) was determined from the cyclic voltammgrams based on the Randles–Sevcik equation^22^:

2 $$j_p = 0.4463nFC_{\rm{cat}}\left( {\frac{{nFvD}}{{RT}}} \right)^{\frac{1}{2}},$$

Here *j*_p_ is the peak current density of quasi-reversible redox couple, *n* is the number of electrons transferred in the redox event, *F* is the Faraday’s constant, *C*_*cat*_ is **1**’s concentration, *v* is the scan rate, *R* is the gas constant, and *T* is the temperature of experiment. As derived from Supplementary Fig. 1a, b, *D* = 2.18 × 10^−7^ cm^2^ s^−1^ for species **1** in the electrolyte.

The observed TOF of bulk electrolysis was determined based on the following equation^19,23^:

3 $${\rm{TOF}} = \left( {\frac{{j_{{\rm{product}}}}}{{nFC_{\rm{cat}}}}} \right)^2\frac{1}{D},$$

Here, *j*_product_ is the partial current density of product formation in bulk electrolysis, *n* is the number of electrons required to generate one product molecule, *F* is the Faraday’s constant, *C*_cat_ is the concentration of catalyst **1**, *D* is the diffusion coefficent of catalyst (2.18 × 10^−7^ cm^2^ s^−1^ for species **1** as determined above).

Similarly, the TON of bulk electrolysis was determined based on the following equation^19,23^:

4 $${\rm{TON}} = \frac{{C_{\rm{product}}V_{\rm{solution}}}}{{AC_{\rm{cat}}}}\sqrt {\frac{{\rm{TOF}}}{D}}.$$

Here, *C*_product_ is the product concentration after bulk electrolysis, *V*_solution_ is the total electrolyte volume, *A* is the electrode area, *C*_cat_ is the concentration of catalyst **1**, *D* is the diffusion coefficent of catalyst (2.18 × 10^−7^ cm^2^ s^−1^ for species **1** as determined above), and TOF is the TOF calculated based on above protocol.

On the condition of a homogenous process with molecular catalyst **1**, the diffusion coefficient extracted from the V(V)/V(IV) redox couple is a reasonable approximate of the real catalytic redox couple, the vanadium(V)-oxo dimer and its one-electron-deficient cation radical, because we are unable to characterize the cation radical due to its transient nature. Here, we offer a rough estimate of the relative uncertainty of such an approximation based on our proposed mechanism. The Stokes–Einstein relationship^41^ predicts that the diffusion coefficient *D* of similar molecules follows: *D* ~MW^−1/3^, in which MW is the molecular weight. As MW = 538.16 g mol^−1^ for **1**, the relative uncertainty of *D* in our procedure is about 6% or 12%, assuming the loss of one or even two of the sulfonic ligands, respectively. As the TOF ~*D*^−1^^23^, at most about 10% relatively uncertainly will incur in our practice.

### Computational methods

All calculations were performed with Turbomole^42–52^ using the M06 density functional^53^. The def2-SVP basis set was used for geometry optimizations and free energy corrections, and the def2-TZVP basis set was used for electronic energies^54^. Solvation was modeled with COSMO^55^ with the dielectric constant set to 101^56^. Images were rendered using Chemcraft^57^. The natural bond orbital (NBO) analysis was used for atomic charge calculations^36^.

### Details of XAS experiments

XAS, including X-ray absorption near edge spectra (XANES) and extended X-ray absorption fine structure (EXAFS), at V K-edge were collected in total-fluorescence-yield mode at ambient conditions at BL17C of National Synchrotron Radiation Center (NSRRC), Hsinchu, Taiwan. Spectra were recorded in the energy range from −100 to 600 eV, relative to that of V K-edge absorption (5465.0 eV). The XAS spectra were processed by subtracting the baseline of pre-edge and normalizing that of post-edge. EXAFS analysis was carried out using Fourier transform on k^3^-weighted EXAFS oscillations to assess the contribution of each bond pair to Fourier transform peak. The curve fitting of EXAFS spectra was conducted using the software, REX2000, with FEFF program.

The operando XAS experiments at V K-edge were conducted under the same procedure at TPS beamline 44A of NSRRC, Hsinchu, Taiwan. A three-electrode arrangement was used during the operando measurements. The electrolyte was saturated with 1-bar CH_4_, and the measurements were performed using an Autolab PGSTAT204 potentiostat (Metrohm Autolab) in a customized reactor.

### Operando Raman characterization

A three-electrode setup in a home-made cell was adopted for Operando Raman spectroscopy and the electrochemical measurements. The measurements in a CH_4_-saturated electrolyte were recorded using a Raman microscopy (UniNano UNIDRON) and an Autolab PGSTAT204 potentiostat (Metrohm Autolab). A laser of 633 nm with a spot size of ~1 µm^2^ served as the excitation source, and the output power was 2.5 mW. A 50× objective lens was employed for operando measurements during electrolysis, while all results were obtained under an exposure duration of 3 s with the accumulation number of 60 times.

## Supplementary information

Supplementary Information

## Data Availability

The data that support the findings of this study are available from the corresponding author upon reasonable request. The source data underlying Figs. 2a, d–f, 3, and Supplementary Figs. 1a, b, 2c, 6c, 7, 9c, 11c, 11d, 13a, 15, 16, 17c, d are provided as Source Data file following the url: 10.6084/m9.figshare.12525524.
