# Supplementary material for: Ambient methane functionalization initiated by electrochemical oxidation of a vanadium (V)-oxo dimer
Source: Nat Commun. 2020 Jul 23;11:3686. doi: 10.1038/s41467-020-17494-w (PMC7378254; doi:10.1038/s41467-020-17494-w)
Supplement: Supplementary file 1 — Supplementary Information [file 41467_2020_17494_MOESM1_ESM.pdf]

Supplementary Information

Ambient methane functionalization initiated by electrochemical oxidation of a vanadium (V)-oxo dimer

Deng, *et al.*

Supplementary Figures

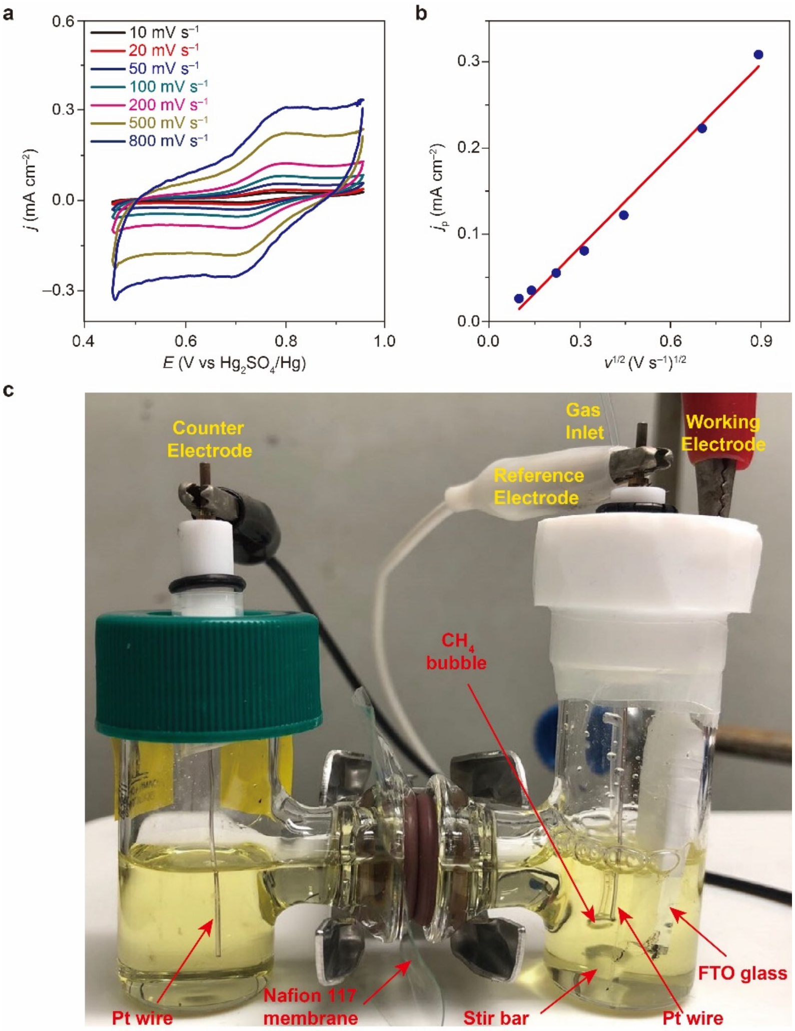

Supplementary Fig. 1 Procedure to obtain the catalyst's diffusion coefficient and the bulk-electrolysis setup. a, Cyclic voltammograms of 1 mM **1** at different scan rates on Pt working electrode. **b**, The current densities of anodic peaks (j_p) versus the square roots of scan rates (v). The linear correlation between j_p and $v^{1/2}$ determines the diffusion coefficient D for species **1**. **c**, Illustration of the two-chamber electrochemical setup for the bulk-electrolysis experiment under ambient CH_4 pressure. Nafion 117 membrane as the separator, a piece of commercial fluorine-doped tin-oxide (FTO) glass as the working electrode, two Pt wires as the pseudo-reference and counter electrodes, respectively.

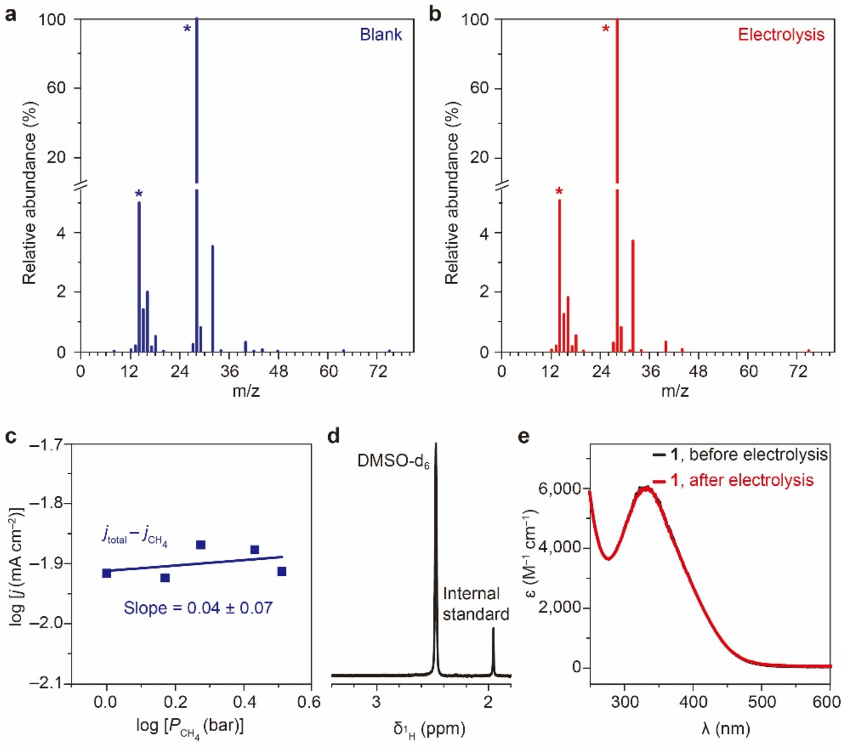

Supplementary Fig. 2 Other possible gas or liquid products and the catalyst's stability for electrochemical CH_4 functionalization. **a**, **b**, MS results of the gaseous samples before (**a**) and during (**b**) the electrolysis in CH_4. The spectra were monitored at a retention time of ~3.3 min by GC-MS. *, peaks correspond to the presence of N_2. H_2S, SO_2, CO and CO_2 as possible gas products were not observed within our detection limit. **c**, The logarithmic of current density that does not active CH_4, log(j_{total}−j_{CH4}) versus the logarithmic of CH_4 pressure, log(p_{CH4}). **d**, ^{1}H NMR spectrum of liquid sample after a 6-hr bulk electrolysis when E = 1.855 V vs. Hg_2SO_4/Hg. **e**, Molar absorption coefficients of **1**'s solution before (black) and after (red) a 6-hr electrolysis (E = 2.105 V vs. Hg_2SO_4/Hg). The absorption spectra were recorded after a 25-time dilution in order to avoid saturating the detector. Unless noted specifically, 25 ºC, 10 mM **1** in 98% H_2SO_4, E = 2.255 V vs. Hg_2SO_4/Hg, p_{CH4} = 1 bar.

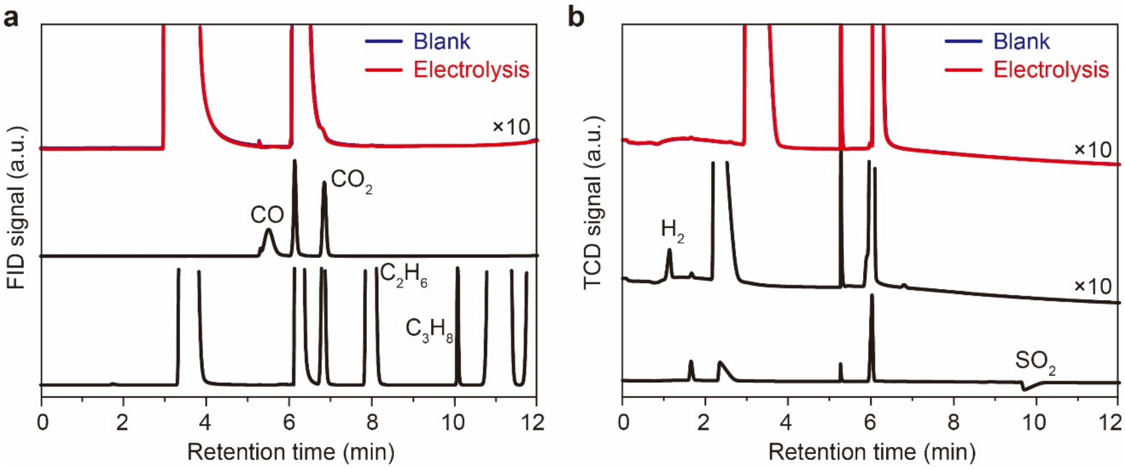

Supplementary Fig. 3 Detection of gas effluent via gas chromatography. Gas chromatographs of flame-ionization detector (**a**) and thermal-conductivity detector (**b**) for the downstream gas effluents before (blue) and during the electrocatalysis (red). Black traces, standard samples. ×10, signal amplified by a factor of 10. 25 ºC, 10 mM **1** in 98% H_2SO_4, E = 2.255 V vs. Hg_2SO_4/Hg, p_{CH4} = 1 bar.

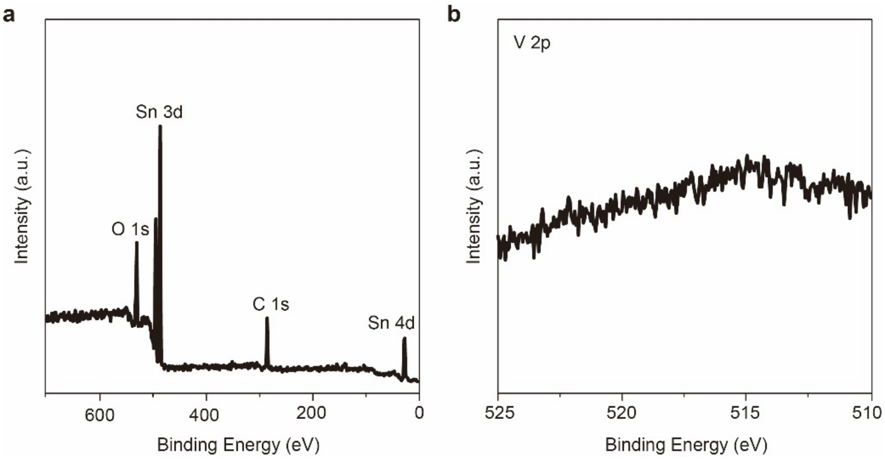

Supplementary Fig. 4 X-ray photoelectron spectroscopy (XPS) results of FTO electrode after electrolysis. Wide scan XPS spectrum (**a**) and V 2p XPS spectra (**b**) of a FTO electrode after 6-hr electrolysis with 10 mM **1** detected no residual V signal on the electrode despite the observation of CH_3OSO_3H formation. 1-bar CH_4, E = 2.255 V vs. Hg_2SO_4/Hg.

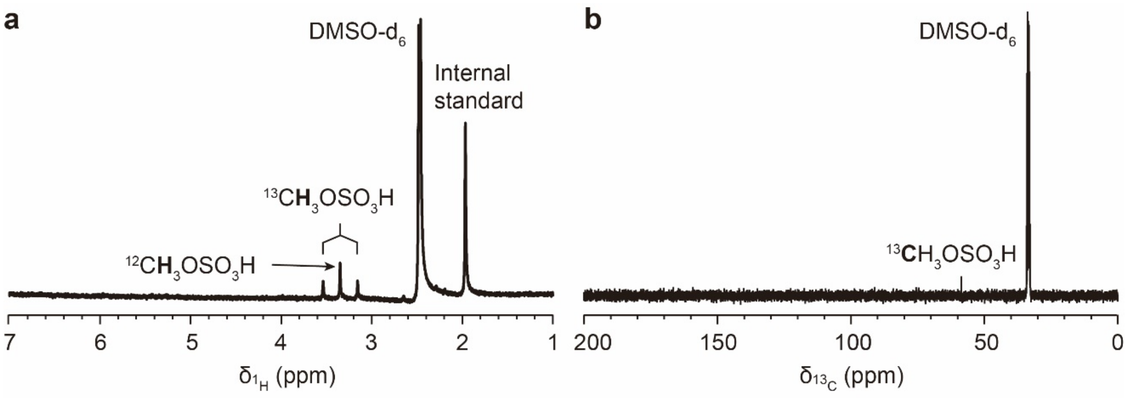

Supplementary Fig. 5 Experiment of isotople labeling with 50% ^{13}C-enriched CH_4. ^{1}H NMR (**a**) and ^{13}C NMR (**b**) spectra of liquid samples after 6-hr electrolysis with 50% ^{13}C-enriched CH_4. No other liquid products except CH_3OSO_3H was observed within our detection limit. Acetic acid (CH_3COOH) was used as internal standard. 25 ºC, 10 mM **1** in 98% H_2SO_4, E = 2.255 V vs. Hg_2SO_4/Hg, p_{CH4} = 1 bar.

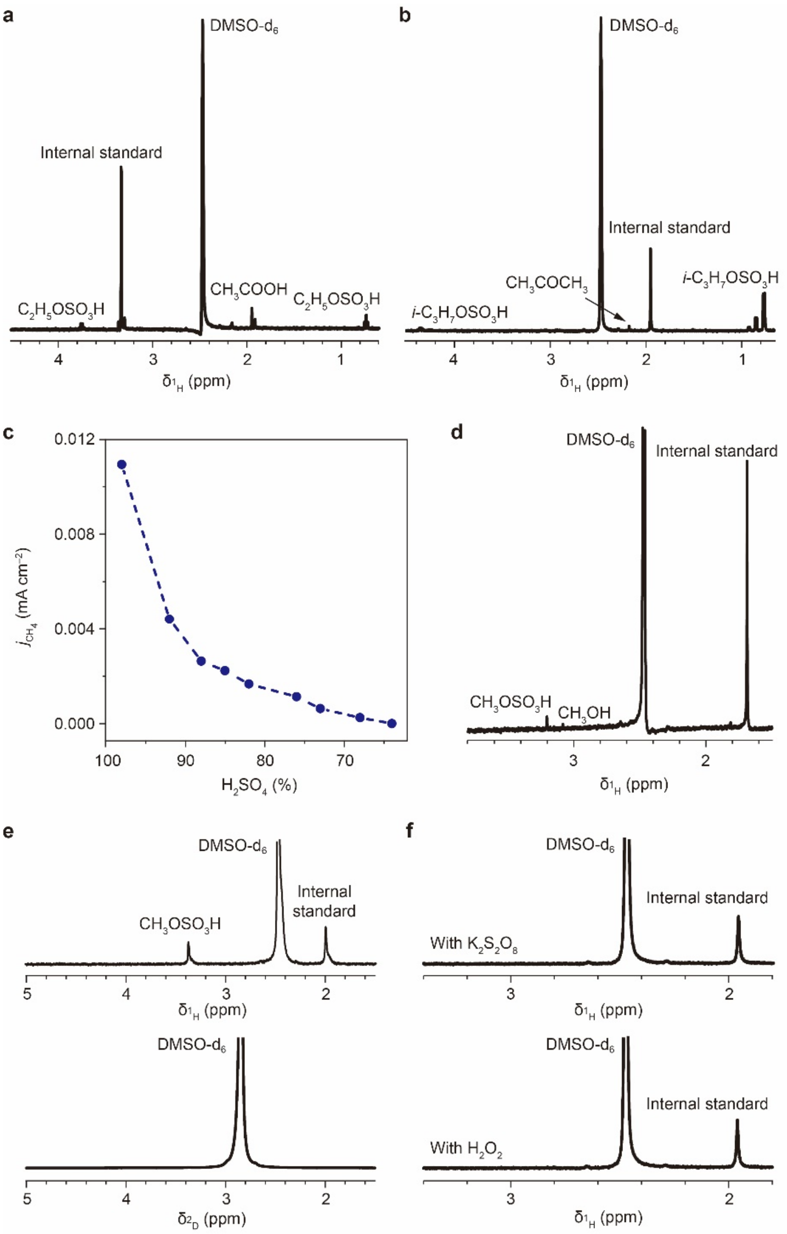

Supplementary Fig. 6 Catalysis with different substrates, acid concentrations, deuterated solvents, and other control experiments. **a**, **b**, ^{1}H NMR spectrum of liquid aliquots after bulk electrolysis under 1-bar C_2H_6 (**a**) and 1-bar C_3H_8 (**b**). Methyl bisulfate (CH_3OSO_3H) and acetic acid (CH_3COOH) as the internal standards in **a** and **b**, respectively. 25 ºC, 0.7 mM **1**, E = 2.255 V vs. Hg_2SO_4/Hg. **c**, Current density for CH_4 functionalization (j_{CH4}) versus the H_2SO_4 concentration. **d**, ^{1}H NMR spectrum of liquid samples after bulk

electrolysis in 85% H_2SO_4. A mixture of CH_3OSO_3H and CH_3OH were detected as the product of CH_4 functionalization. **c**, **d**, 25 ºC, 0.7 mM **1**, 1-bar CH_4, E = 2.2 V vs. Hg_2SO_4/Hg. **e**, ^{1}H NMR (upper) and ^{2}D NMR (lower) spectra of liquid aliquots after 6-hr electrolysis in 98% D_2SO_4. 25 ºC, 10 mM **1**, 1-bar CH_4, E = 2.255 V vs. Hg_2SO_4/Hg. Acetic acid (CH_3COOH) was used as internal standard for the experiment of ^{1}H NMR. The extent of H/D exchange in the reaction, if any, is below the detection limit. **f**, ^{1}H NMR spectra of liquid samples after a 6-hr exposure of CH_4 at 25 ºC to a mixture of 10 mM **1** and 10 mM $K_2S_2O_8$ (upper), as well as to a mixture of 10 mM **1** and 10 mM H_2O_2 (lower). Acetic acid (CH_3COOH) was used as internal standard. The formation of methyl bisulfate (CH_3OSO_3H) was not observed within our detection limit.

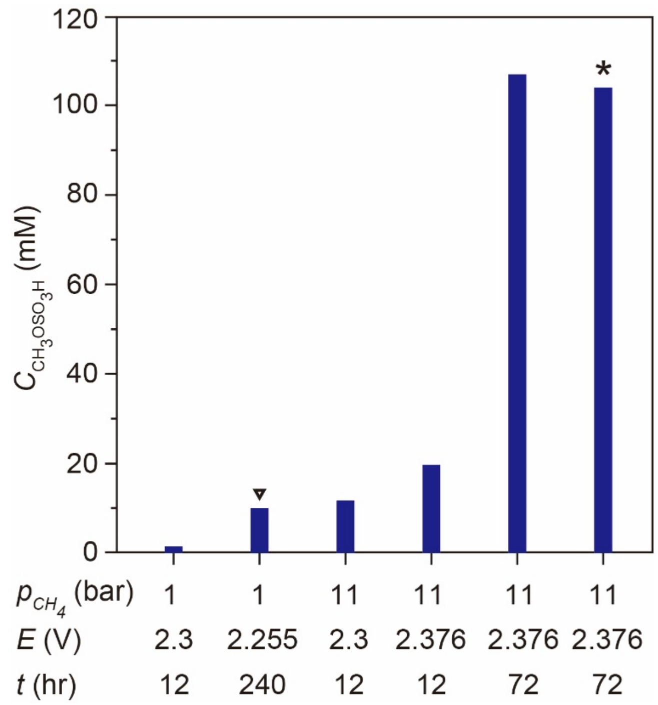

Supplementary Fig. 7 Concentrations of yielded CH_3OSO_3H ($C_{CH3OSO3H}$) from electrochemical CH_4 functionalization under different reaction conditions. p_{CH4}, pressure of CH_4 (bar); *E*, electrochemical potential (V vs. Hg_2SO_4/Hg); *t*, electrolysis duration (hr). ▼, 0.7 mM **1**, natural gas mixture supplied by SoCalGas; *, with pre-added 1 M CH_3OSO_3H. 25 ºC, 50 mM **1** in 98% H_2SO_4 unless mentioned specifically.

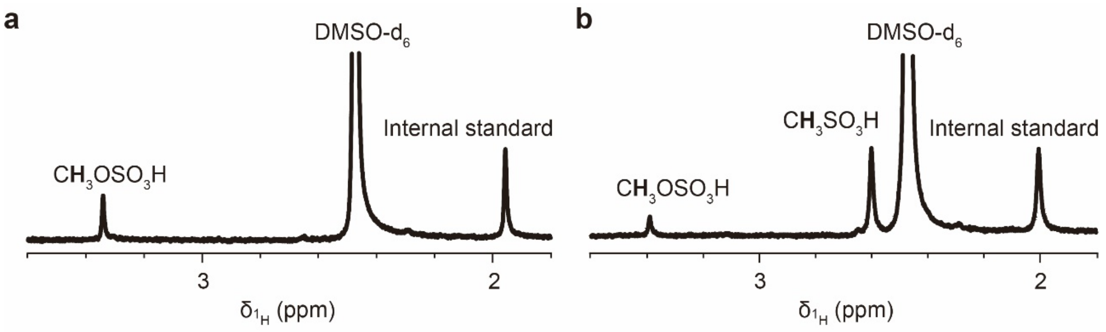

Supplementary Fig. 8 CH_3SO_3H formation in the presence of SO_3. ^{1}H NMR spectra of liquid aliquots after 6-hr electrolysis in 98% H_2SO_4 (**a**) and oleum (20% SO_3 in H_2SO_4) (**b**). 25 ºC, 10 mM **1**, 1-bar CH_4. Acetic acid (CH_3COOH) was used as internal standard for the experiment of ^{1}H NMR. E = 2.255 V and 2.363 V vs. Hg_2SO_4/Hg for **a** and **b**, respectively.

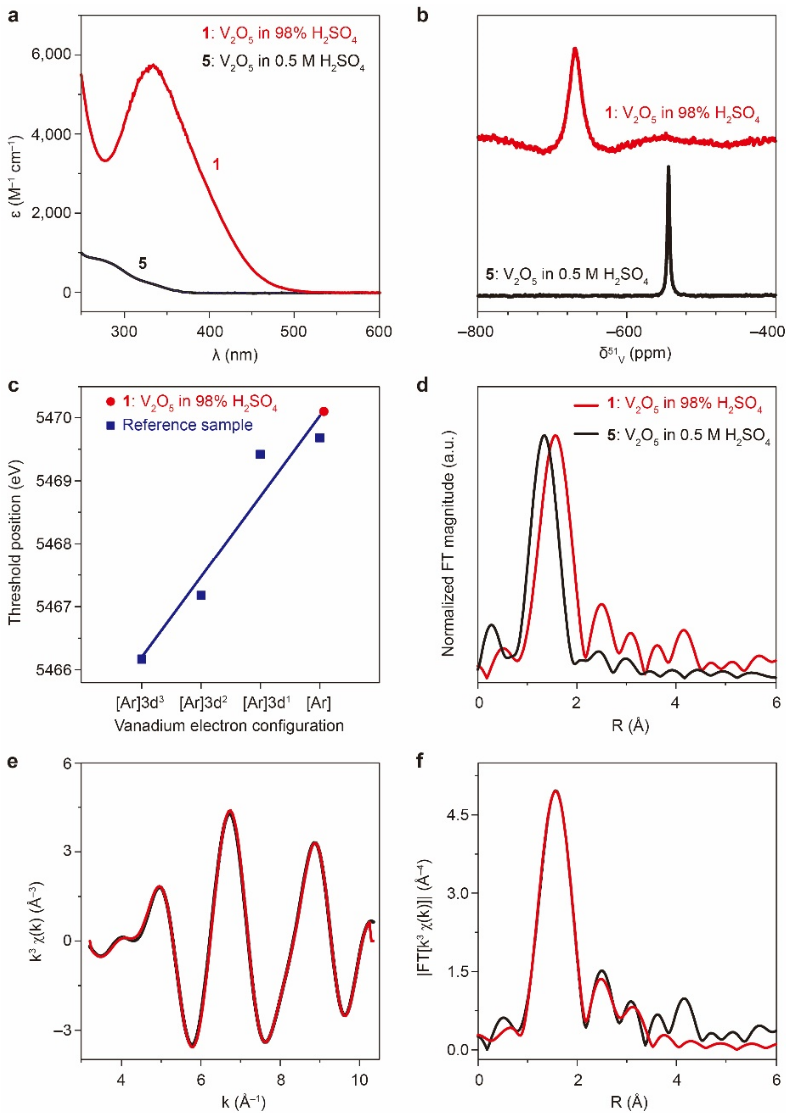

Supplementary Fig. 9 Characterizations of the catalyst via optical absorption, ^{51}V NMR, and X-ray absorption spectroscopy. a, **b**, Molar absorption coefficients (**a**) and ^{51}V NMR spectrum (**b**) of catalyst **1** (red), prepared by dissolving V_2O_5 in 98% H_2SO_4. The monometallic VO_2^+ species (**5**, black), prepared by dissolving V_2O_5 in 0.5 M H_2SO_4, was displayed for comparison. 0.4 mM and 10 mM of vanadium in **a** and **b**, respectively. **c**, Threshold position versus vanadium electron configuration in V K-edge XANES spectra of VO, V_2O_3, VO_2, V_2O_5 and 10 mM **1** in 98% H_2SO_4. **d**, Extended X-ray absorption fine

structure (EXAFS) of 10 mM **1** in 98% H_2SO_4 as well as 10 mM monometallic VO_2^+ species (**5**) in 0.5 M H_2SO_4. **e**, **f**, The EXAFS *k*-space (**e**) and *R*-space (**f**) curves for the experimental results of catalyst **1** (black) as well as the corresponding numerical fitting (red). The parameters that lead to such a fitted curve are displayed in Fig. 4d.

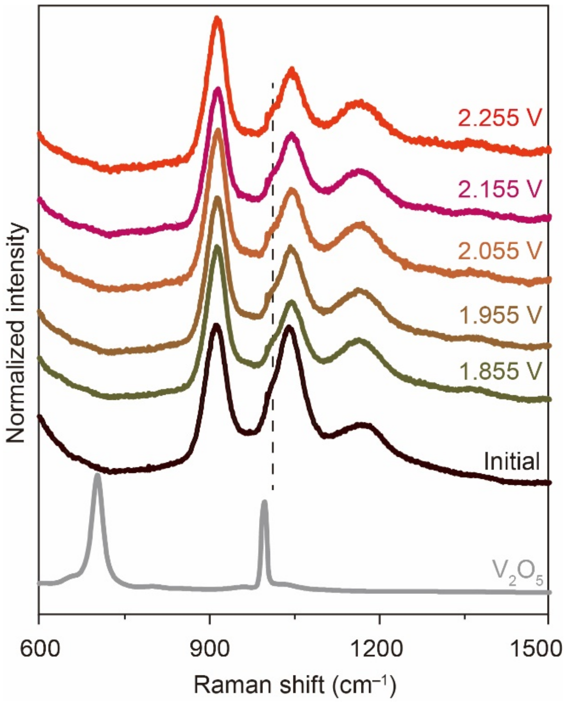

Supplementary Fig. 10 Operando Raman spectroscopy. Raman spectra for **1** before electrolysis (initial) and during electrolysis under 1-bar CH_4 at different electrode potentials *E*. Spectrum of solid V_2O_5 was presented for comparison. Dashed lines indicates the characteristic 988 cm^{-1} vibration of **1** reported in previous literature[1]. The other major peaks originates from the solvent. 25 ºC, 100 mM **1** in 98% H_2SO_4.

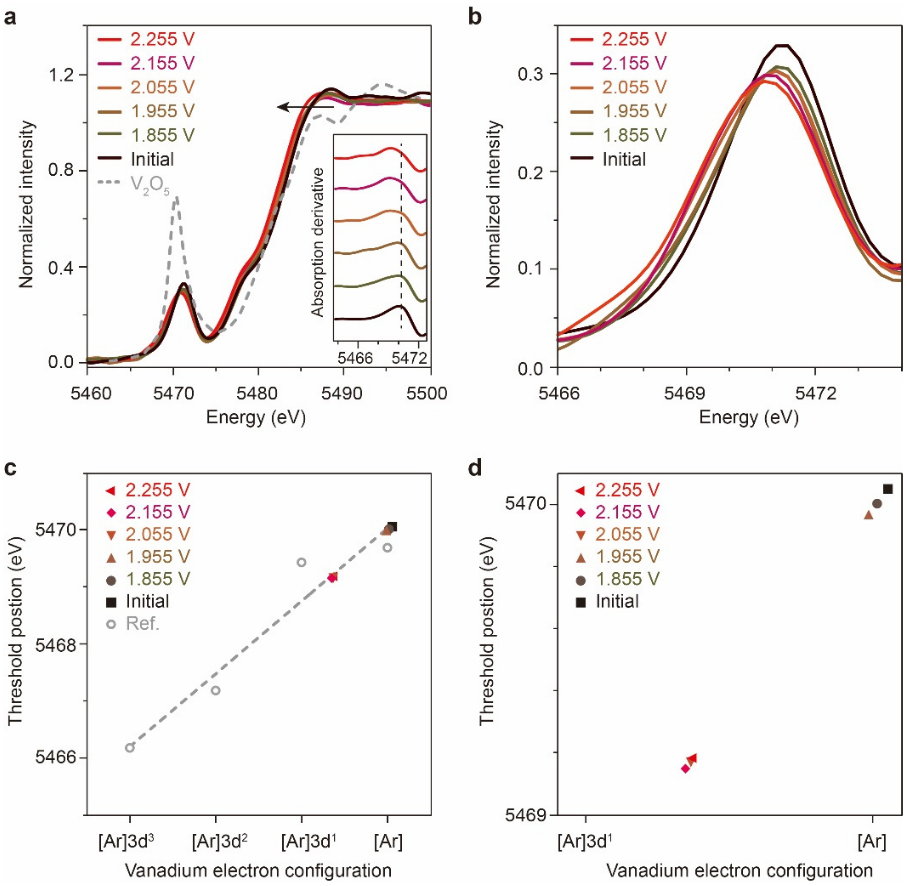

Supplementary Fig. 11 Operando spectra of X-ray absorption near-edge structure (XANES). Normalized intensity of V K-edge XANES spectra (**a**) and the magnified spectra at pre-edge energy range (**b**) for vanadium (V)-oxo dimer before electrolysis (initial) and during electrolysis at different electrode potentials (vs. Hg_2SO_4/Hg). 25 ºC, 1-bar CH_4, 50 mM catalyst **1** in 98% H_2SO_4. Spectrum of solid V_2O_5 sample (dashed grey) is also presented for comparison. Inset of **a** is first derivative of the XANES spectra. **c**, **d**, Threshold position versus vanadium electron configuration in V K-edge XANES spectra. Reference samples (Ref., grey open circle) of VO, V_2O_3, VO_2, and V_2O_5 were presented for comparison.

a

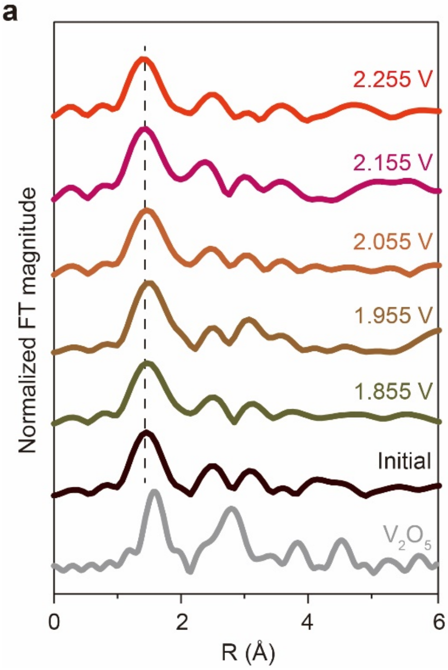

b

Fitting results from EXAFS

E (V)	Shell	C. N.	R (Å)
Initial	V–O	1.2 (0.2)	1.53 (0.03)
	V–O	1.1 (0.2)	1.68 (0.05)
	V–O	3.0 (0.4)	2.07 (0.07)
1.855	V–O	1.2 (0.2)	1.62 (0.05)
	V–O	1.1 (0.3)	2.05 (0.07)
	V–O	3.1 (0.7)	2.69 (0.08)
1.955	V–O	1.2 (0.3)	1.64 (0.05)
	V–O	1.1 (0.2)	2.03 (0.06)
	V–O	3.0 (0.4)	2.68 (0.08)
2.055	V–O	1.0 (0.2)	1.61 (0.05)
	V–O	1.1 (0.3)	2.00 (0.06)
	V–O	2.7 (0.5)	2.65 (0.08)
2.155	V–O	1.1 (0.2)	1.63 (0.04)
	V–O	1.2 (0.3)	1.98 (0.05)
	V–O	2.8 (0.4)	2.63 (0.08)
2.255	V–O	1.2 (0.3)	1.73 (0.06)
	V–O	1.3 (0.4)	1.95 (0.05)
	V–O	2.6 (0.7)	2.61 (0.08)

Supplementary Fig. 12 Operando spectra of Extended X-ray absorption fine structure (EXAFS). a, Normalized intensity of EXAFS results for catalyst **1** before electrolysis (initial) and during electrolysis at different electrode potentials (vs. Hg_2SO_4/Hg). 25 ºC, 1-bar CH_4, 50 mM catalyst **1** in 98% H_2SO_4. Spectrum of solid V_2O_5 sample (grey) was also presented for comparison. **b**, Calculated coordination number (C. N.) and the distance (R) away from V atom based on spectra in **a**. The red entries denote the C. N. and R values of V-O single bond in the sulfonic ligands. A decrease of average number of sulfonic ligands was concurrent with increasing *E* values.

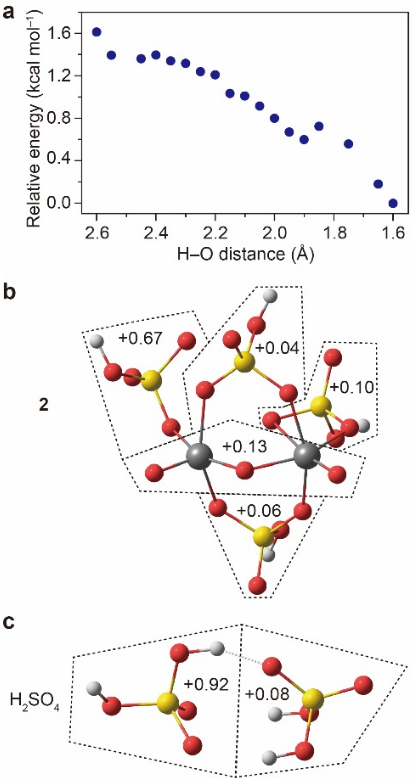

Supplementary Fig. 13 DFT calculations of reaction trajectory and the changes of atomic charges. **a**, Potential energy surface, plotted by the H−O bond distance, during the CH_4 activation step between CH_4 and **2**. **b**, **c**, Changes in atomic charges between **1a** and cation radical **2** (**b**), as well as a sulfuric acid molecule and its oxidized variant after one-electron removal (**c**).

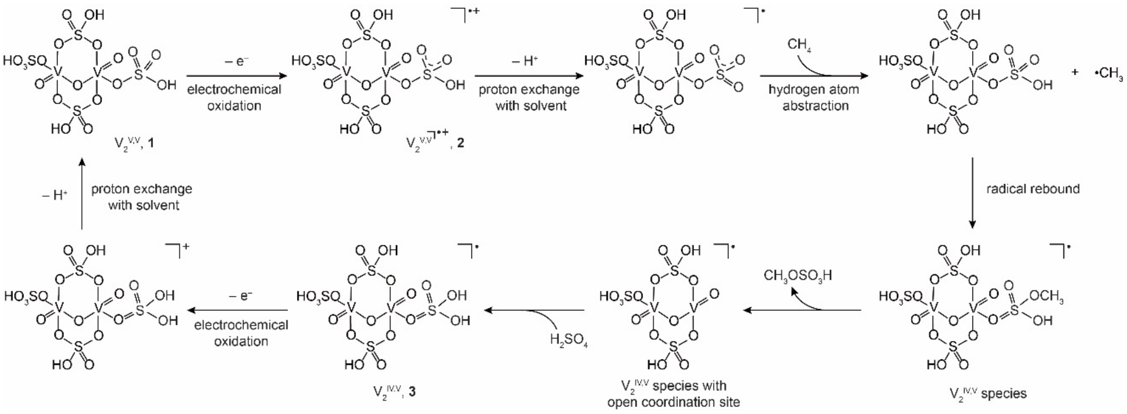

Supplementary Fig. 14 A more detailed, step-by-step mechanism. The presented mechanism is tentatively proposed for our catalytic system. It remains unclear experimentally whether a deprotonation step precedes the H-atom abstraction.

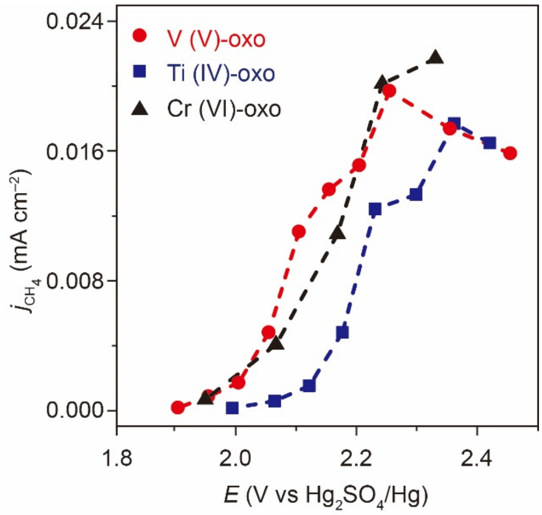

Supplementary Fig. 15 Reactivities of other d^0 metal-oxo species for ambient electrocatalytic CH_4 functionalization. Current density for CH_4 functionalization (j_{CH4}) versus electrode potential E with 10 mM vanadium (V)-oxo (**1**), titanium (IV)-oxo, and chromium (VI)-oxo species, respectively. 25 ºC, 1-bar CH_4, 98% H_2SO_4.

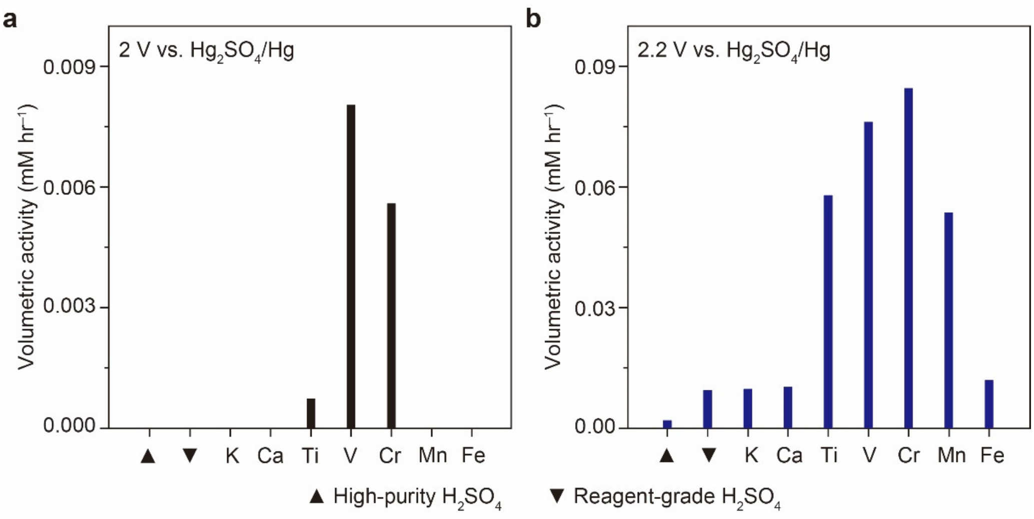

Supplementary Fig. 16 Reactivities of the first half of Period 4 elements. Rate of CH_3OSO_3H formation at E = 2 V (**a**) and 2.2 V (**b**) vs. Hg_2SO_4/Hg in 98% H_2SO_4. ▲ & ▼, high-purity and reagent-grade 98% H_2SO_4 without added metal salts, which contains 0.3 and 5 ppm of metal impurities, respectively. 10 mM of KCl, $CaSO_4$, $TiOSO_4$, V_2O_5, K_2CrO_4, $KMnO_4$, and $FeCl_2$ were dissolved in reagent-grade 98% H_2SO_4. 25 ºC, 1-bar CH_4. Sc was not studies as we were unable to find a simple salt or oxide that has a solubility of higher than 10 mM in 98% H_2SO_4.

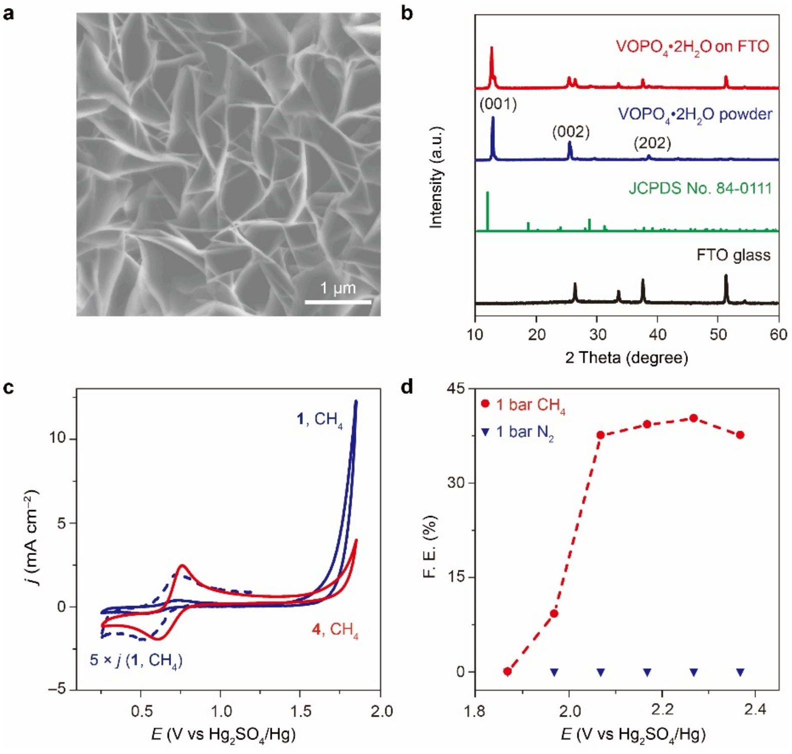

Supplementary Fig. 17 A heterogeneous variant of the reported electrocatalyst. a, Image of scanning electron microscopy of $VOPO_4{\cdot}2H_2O$ (**4**), the heterogeneous variant of the reported catalyst. **b**, Powder X-ray diffraction patterns of as-prepared **4** (blue), **4** loaded on FTO electrode (red), the standard pattern of $VOPO_4{\cdot}2H_2O$ (green, JCPDS No. 84-0111), and the blank FTO electrode as a control (black). **c**, Cyclic voltammograms of 10 mM **1** (blue) and loaded **4** with a loading amount of 1.9 mg cm^{-2} (red). 25 ºC, 98% H_2SO_4, 100 mV s^{-1}, 1-bar CH_4, 2-mm Pt working electrode. The current density of **1** is magnified by a factor of 5 (blue dashed line). **d**, Faradaic efficiency (F. E.) of catalyst **4** as a function of electrode potential E in 1-bar CH_4 (red) and 1-bar N_2 (blue). 25 ºC, 98% H_2SO_4, data recorded from 6-hr bulk electrolysis.

Supplementary Tables

Supplementary Table 1 Reaction conditions, turnover frequencies and activation energies of catalytic CH_4 functionalization

Catalysts	Solvent	Oxidant/ reactant	Temperature (ºC)	P_{CH4} (bar)	Product	TOF [a] (hr^{-1})	E_a [b] (kcal mol^{-1})	References
**1** [c]	98% H_2SO_4	Echem [d]	25	1	CH_3OSO_3H	483	10.8 [e]	*This work*
**1** [c]	98% H_2SO_4	Echem [d]	25	3	CH_3OSO_3H	1336	10.8 [e]	*This work*
MMSP or DMSP [f]	20 ~ 60% SO_3 / H_2SO_4	SO_3	50	~100	CH_3SO_3H	–	~26.5	[2]
Cp*$_2$ScMe [g]	Cyclohexane	$CH_3CH=CH_2$	80	– [h]	$(CH_3)_3CH$	–	11.4	[3]
(NHC-pym)$PdCl_2$ [i]	CF_3COOH	$K_2S_2O_8$	90	30	CH_3OCOCF_3	~2.4	39.5 [j]	[4]
$PdSO_4$	20% SO_3 / H_2SO_4	Echem [k] and SO_3	70	~34	CH_3OSO_3H & CH_3SO_3H	~0.2 [l]<br>~1.9 [l]	–<br>–	[5]<br>[5]
$PdSO_4$	Concentrated H_2SO_4	Echem [m]	140	~34	– [n]	2000	25.9	[5]
K_2PtCl_4-Na_2PtCl_6	0.5 M H_2SO_4	Echem [o]	130	~47	CH_3X [p]	0.29	–	[6]
$[Cu^ICu^ICu^I$(7-N-Etppz)$]^{1+}$ [q]	CH_3CN	O_2	0	1	CH_3OH	~2.8	–	[7]
$HgSO_4$	100% H_2SO_4	H_2SO_4	180	~35	CH_3OSO_3H	~3.6	34 [r]	[8]
$PdSO_4$	96% H_2SO_4	H_2SO_4	180	~27	CH_3COOH	~3.6	27.9 [s]	[9]
$Au_2(SO_4)_3$	96% H_2SO_4	H_2SeO_4	180	27	CH_3OSO_3H	~3.6	30	[10]
Ir(COD)Cl/dmpe [t]	Cyclohexane	B_2Pin_2 [u]	150	~34	CH_3Bpin [v]	~6.5	25.9	[11]
(bpym)$PtCl_2$ [w]	102% H_2SO_4	SO_3	220	34	CH_3OSO_3H	~36	36 [x]	[12]
$Ce(OTf)_4$ [y]	CH_3CN	CCl_3CH_2OH	25	50	Aryl and alkyl derivatives	~161	–	[13]
(bpym)$PtCl_2$ [w]	20% SO_3 / H_2SO_4	SO_3	215	~65	CH_3OSO_3H	1280	–	[14]
K_2PtCl_4	20% SO_3 / H_2SO_4	SO_3	215	~65	CH_3OSO_3H	23400	–	[14]

[a] TOF, turnover frequency. [b] E_a, apparent activation energy. [c] Catalyst concentration is 0.7 mM. [d] E = 2.255 V vs. Hg_2SO_4/Hg. [e] The value is obtained at E = 2.055 V vs. Hg_2SO_4/Hg. [f] Using as electrophilic initiator, MMSP, monomethylsulfonylperoxide sulfuric acid $[HOS(O)_2OOS(O)_2CH_3]$; DMSP, bis(methylsulfonyl) peroxide $[H_3CS(O)_2OOS(O)_2CH_3]$. [g] Cp*, cyclopentadiene. [h] Not specified, it should be ambient pressure. [i] NHC-pym, N-heterocyclic carbene-pyrimidine. [j] Value is obtained from Ref. [15]. [k] E = 2.0 V vs. Ag_2SO_4/Ag. [l] Calculated by $PdSO_4$ concentration. [m] Step potentials of 2.0 V followed by 0.5 V vs. Ag_2SO_4/Ag. [n] Proposed yet not experimentally quantified in this specific experiment. [o] Average current is 1.19 mA for 4.9 h. [p] CH_3X represents the product of CH_3OH and CH_3Cl, products $CH_2(OH)_2$, HCOOH, and CO_2 are not included here. [q] 7-N-Etppz, 3,3'-(1,4-diazepane-1,4-diyl)bis[1-(4-ethylpiperazine-1-yl)propan-2-ol]. [r] Value is obtained from Ref. [16]. [s] Value is obtained from Ref. [17]. [t] COD, 1,5-cyclooctadiene; dmpe, 1,2-bis(dimethylphosphino)ethane. [u] B_2Pin_2, 4,4,4',4',5,5,5',5'-Octamethyl-2,2'-bi-1,3,2-dioxaborolane. [v] CH_3Bpin, 2,4,4,5,5-pentamethyl-1,3,2-dioxaborolane. [w] bpym, bipyrimidine. [x] Value is obtained from Ref. [18]. [y] Photocatalysis, OTf, trifluoromethanesulfonate.

Supplementary Table 2 The turnover numbers and Faradaic efficiencies with natural gas as the substrate [a]

	C_1 product	C_2 products		C_3 products		Total [b]
	CH_3OSO_3H	CH_3COOH	$C_2H_5OSO_3H$	CH_3COCH_3	*i*-$C_3H_7OSO_3H$	
TONs [c]	106,883	8,934	405	216	– [d]	116,438
F. E. (%) [e]	43.8	38.0	2.6	3.9	– [d]	88.3

[a] 25 ºC, 0.7 mM **1** in 98% H_2SO_4, 1-bar natural gas supplied to UCLA by SoCalGas, *E* = 2.255 V vs. Hg_2SO_4/Hg, 240-hr bulk electrolysis. [b] Trace amount of products with more than 3 carbons (> C_3) were observed yet not included here. [c] TON, turnover number. The cumulative values after 240-hr electrolysis are reported. [d] Not detected from ^{1}H NMR. [e] F. E., Faradaic efficiencies. The reported values are 240-hr averages.

Supplementary Table 3 Comparison of structural information between EXAFS data and DFT calculations [a]

Results from the analysis of EXAFS data [b]			Values from DFT calculations						
			**1a**				**1b**		
Shell	C. N.	R (Å)	Shell	C. N.	R_1 (Å)	R_2 (Å)	Shell	C. N.	R (Å)
V–O	1.1 (0.1)	1.58 (0.01)	V=O	1	1.53	1.53	V=O	1	1.53
V–O	1.2 (0.1)	1.68 (0.02)	V–O	4	1.73	1.82	V–O	4	1.76
V–O	3.0 (0.1)	1.96 (0.01)			1.92	1.83			1.86
					1.96	1.93			2.01
V–S	1.0 (0.4)	2.73 (0.05)			2.01	1.98			2.05
V–S	2.0 (0.2)	3.13 (0.07)	V–S	3	2.82	3.17	V–S	2	2.67
					3.19	3.19			2.85
V–V	1.0 (0.2)	3.27 (0.05)			3.20	3.23	V–V	1	3.38
			V–V	1	3.14	3.14			

[a] C. N., coordination number; R, distance away from the V atom. [b] EXAFS data recorded for 10 mM **1** in 98% H_2SO_4.

Supplementary Notes

Supplementary Note 1

In this section we aim to discuss the mathematical derivation of our TOF analysis, the implicit underestimation of our analysis, and the implication of those reported TOF values for other electrochemical data presented in this manuscript.

Part 1: mathematical derivation of our TOF analysis employed in the study

In the following we will derive the Tafel slope, *i.e.* the electrochemical potential (*E*) needed for electrocatalytic current density (*i*) to increase by one order of magnitude at steady state, based on two different assumptions for homogenous electrochemical catalysis.

$$Tafel\ slope \equiv \frac{\partial E}{\partial log_{10}(i)} \quad (1)$$

If we define the electrochemical overpotential $\eta \equiv E - E_{X/A}$, in which $E_{X/A}$ is the thermodynamic redox potential for the X/A turnover, the Tafel slope can be rewritten as:

$$Tafel\ slope \equiv \frac{\partial \eta}{\partial log_{10}(i)} \quad (2)$$

In particular, we would like to discuss the redox-mediating scheme, in which an

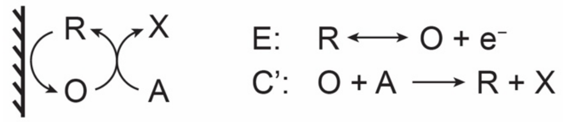

electrochemical charge transfer (E step) is followed by a solution homogenous chemical reaction (C' step) that regenerates the redox mediator as shown in the scheme on the right. There are two general assumptions for the following derivations. (1) we assume that only a single-electron transfer is involved for one catalytic turnover for the sake of derivation simplicity. It can be proved and has been shown that the conclusion derived here remains valid for multi-electron processes as long as either the first E step is turnover-limiting or

the net E steps are fast, *i.e.* outer-sphere processes[19]. (2) we will confine our derivation to the scenario with relatively small E versus $E_{R/O}$, the redox potential of the R/O couple. This restricts our discussion before the catalytic turnover frequency (TOF) reaches its maximum when all of the redox-mediator are quickly converted to its oxidized form for catalytic turnover[19,20].

Part 1.1: Tafel slope when the C' step is the turnover-limiting step (TLS)

In the classic EC' mechanism, the E step is assumed as out-sphere and the C' step the turnover-limiting step (TLS). Under such assumptions, the net rate of turnover, *i.e.* the catalytic current density, can be expressed below by assuming a model of reaction-diffusion layer as previously reported:[19]

$$\frac{i}{F} = \sqrt{kC_A D_{R/O}} \frac{C_{R/O}}{1 + exp\left[-\frac{F}{RT}\left(E - E_{R/O}\right)\right]}$$

$$= \sqrt{kC_A D_{R/O}} \frac{C_{R/O}}{1 + exp\left[-\frac{F}{RT}\left(\eta + E_{X/A} - E_{R/O}\right)\right]} \quad (3)$$

Here k the volumetric kinetic rate constant of the C' step, $D_{R/O}$ the diffusion coefficient of the redox mediator (assuming to be the same for both R and O), C_A the concentration of substrate which is assumed as a constant to exclude its issue of mass transport, and $C_{R/O}$ the net concentration of R and O. We note here that the above expression is slightly different from the one reported before[19]. This is because here we consider an oxidation reaction with one electron transfer per turnover, while previous derivation is in the context of a reduction reaction with two electron transfers per turnover[19].

Under the stated assumptions, the above expression can be re-expressed as:

$$\frac{i}{F} = \sqrt{kC_A D_{R/O}} \cdot C_{R/O} \cdot exp\left[\frac{F}{RT}\left(E_{X/A} - E_{R/O}\right)\right] \cdot exp\left(\frac{F}{RT}\eta\right) \quad (4)$$

The above expression assumes: $exp\left[-\frac{F}{RT}\left(E_{X/A}-E_{R/O}\right)\right]>1$, which is usually satisfied in typical electrocatalysis.

Therefore, in a EC' mechanism,

$$log_{10}(i)=A+\frac{F}{RT\cdot ln10}\eta\ \Rightarrow\ \frac{\partial\eta}{\partial log_{10}(i)}=\frac{RT\cdot ln10}{F}=0.059\,V\,decade^{-1}\quad(5)$$

This proves that the Tafel slope is 59 mV decade^{-1}, or sometimes approximated as 60 mV decade^{-1}, in a EC' mechanism.

Part 1.2: Tafel slope when the E step is the turnover-limiting step (TLS)

In the scenario when the E step other than the C' step is turnover limiting, the above analysis is not valid anymore. This is because we cannot assume a fast, probably out-sphere R/O redox couple that follows the Nernst equation, which is the underlying assumption of the above derivation[19]. Under current scenario when the E step is slow and turnover-limiting, the rate of catalytic turnover is dependent on the rate of electron transfer at the E step, which should follow the Butler-Volmer equation:

$$i=i_0\left[exp\left(\frac{\alpha F}{RT}\eta\right)-exp\left(-\frac{(1-\alpha)F}{RT}\eta\right)\right]\quad(6)$$

Here i_0 is the exchange current density of the O/R redox couple and α is the transfer coefficient. When the overpotential η is large enough to neglect the rate of back reactions (reduction of O back to R), the above equation can be simplified in the Tafel equation:

$$i=i_0 exp\left(\frac{\alpha F}{RT}\eta\right)\quad(7)$$

In a redox-mediating scheme when the E step is the TLS,

$$log_{10}(i)=A+\frac{\alpha F}{RT\cdot ln10}\eta\ \Rightarrow\ \frac{\partial\eta}{\partial log_{10}(i)}=\frac{RT\cdot ln10}{\alpha F}\quad(8)$$

Typical redox reaction possesses an approximate value of 0.5 for α,[20] therefore,

$$\frac{\partial \eta}{\partial log_{10}(i)} = \frac{2RT \cdot ln10}{F} = 0.118\, V\, decade^{-1} \quad (9)$$

This proves that the Tafel slope is 118 mV decade^{-1}, or sometimes approximated as 120 mV decade^{-1}, in a redox-mediating scheme when the E step is the TLS.

Part 1.3: TOF in a redox-mediating scheme when the E step is the TLS

In the following we will shortly comment over the calculations of TOF values in a redox-mediating scheme when the E step is the TLS.

In the EC' mechanism, the values of TOF and subsequently TON for homogenous electrocatalysis are defined by only counting the catalyst molecules within the diffusion-reaction layer[19,20]. Information of the rate constant *k*, as well as the $TOF = kC_A$ for the C' step can be experimentally obtained, starting from the differential equation for the concentration of oxidized redox mediator ([O]) that governs the diffusion-reaction layer:[19]

$$D_{R/O}\frac{d^2[O]}{dx^2} - kC_A[O] = 0 \quad (10)$$

The boundary conditions of the reaction are:

$$[O]_{x=\mu} = 0, \qquad \left(\frac{d[O]}{dx}\right)_{x=\mu} = 0, \qquad [O]_{x=0} = \frac{C_{R/O}}{1 + exp\left[-\frac{F}{RT}\left(E - E_{R/O}\right)\right]} \quad (11)$$

The general solution of the above differential equation is:

$$[O](x) = [O]_{x=0} \cdot exp\left(-\sqrt{\frac{kC_A}{D_{R/O}}}x\right) \quad (12)$$

In a redox-mediating scenario when the E step is the TLS, the diffusion-reaction layer, the rate constant *k* for the C' step, and the general solution of the differential equation remain valid. The difference is the boundary condition at $x = 0$, and subsequently the expression of $[O]_{x=0}$. Other than assuming a constant concentration of [O] (the Dirichlet boundary

condition), here we have a constant flux of [O] from the electrode (the Neumann boundary condition):

$$\left(\frac{d[O]}{dx}\right)_{x=0} = -\frac{i}{FD_{R/O}} \quad (13)$$

Therefore, based on the general solution of the above equation, we can have,

$$\left(\frac{d[O]}{dx}\right)_{x=0} = -\sqrt{\frac{kC_A}{D_{R/O}}}[O]_{x=0} = -\frac{i}{FD_{R/O}} \Rightarrow [O]_{x=0} = \frac{i}{FD_{R/O}}\sqrt{\frac{D_{R/O}}{kC_A}} \quad (14)$$

While $[O]_{x=0}$ cannot be independently fetched in a redox-mediating scenario when the E step is the TLS, what we know for sure is that [O] cannot exceed $C_{R/O}$, the catalyst concentration experimentally added into the solution,

$$[O]_{x=0} < C_{R/O}$$

Therefore, a conservative, under-estimating method of calculating the rate constant k is to assume $[O]_{x=0} = C_{R/O}$ at any given conditions. This will lead to an under-estimated value of TOF,

$$TOF = kC_A = \left(\frac{i}{FC_{R/O}}\right)^2 \frac{1}{D_{R/O}} \quad (15)$$

This is the equation that we applied for the calculation of TOF in the manuscript. It is interesting to note that the TOF calculation at steady state, at least for a conservative, under-estimated value, is the same for both the EC' mechanism and a redox-mediating scenario when the E step is the TLS. Such a similarity originates from the fact that both follows the same model of diffusion-reaction layer.

Part 2: The implication of the estimated TOF values for the other data in the manuscript

The proposed mechanism implies a possibility that the one-electron oxidized intermediate **2** may be electrochemically reduced back to **1** (*E* step) in cyclic voltammetry, indicative in a cathodic redox peak in the reverse scan, before **2** reacts with either CH_4 or solvent (*C'* step). However, this presumed cathodic peak was not observed in either CH_4 or N_2 environment. It suggests that the solution reaction kinetics between **2** and CH_4/solvent are much faster within the time scale of cyclic voltammetry. Such a suggestion is indeed consistent with our underestimated TOF values determined in bulk electrolysis, while additional experiments are needed for verification.

First of all, we would to note that the reported TOF values are underestimated and erred on the side of caution, which may not be suitable for a straightforward interpretation for the data in Fig. 2a.

Second, it is estimated that the TOF values for CH_4 and solvent reaction are about 483 and 150 hr^{-1}, respectively. The TOF value for CH_4 activation at 1-bar condition is reported in the main text and Supplementary Table 1. The TOF value for the reaction between **2** and solvent could be estimated from the *F. E.* values reported in Fig. 2e. With a 90% *F. E.* value and an underestimated TOF value of 1336 hr^{-1} for CH_4 activation at 3-bar CH_4, the TOF rate of solvent oxidation should be about 1336 / 9 ~ 148 hr^{-1}. This suggests that on average it takes about at most 7 and 24 secs for **2** to react with 1-bar CH_4 and solvent, respectively.

Third, the above calculated time constant of reaction with CH_4 and solvent is more or less consistent with the available data. In Fig. 2a, the forward (anodic) and reverse (cathodic) scan in total create a potential window of about 700 mV at a scan rate of 100 mV sec^{-1}. This corresponds to a time scale of about 7 sec. Therefore, roughly within the time scale

of the cyclic voltammetry, about 100% and 30% of the electrochemically generated **2** should have reacted with 1-bar CH_4 and solvent, respectively. Taking into the account that our reported TOF values are underestimated and not all of the soluble oxidized intermediate may diffuse back to the electrode during the scan, there seems a good chance that within a 700-mV potential window at a scan rate of 100 mV sec^{-1} the oxidized species have been fully reacted with the solvent in the absence of CH_4.

Supplementary Note 2

Because a homogenous electrocatalysis with a turnover-limiting *E* step is rare in literature, here we would like to briefly summarize the evidence supporting such a claim and then offers our argument against a few possible criticisms refuting a turnover-limiting *E* step.

Key evidence supporting a turnover-limiting *E* step in our study.

1. Bulk electrolysis displays a Tafel slope of 120 mV dec^{-1} (Fig. 3b), signaling a single electron transfer as the TLS.
2. Operando measurement of XANES data displays a decrease of vanadium's oxidation state in the proximity of electrode's surface (Supplementary Fig. 11). This piece of evidence suggests a molecular catalysis with fast degradation of the oxidant intermediate (see Supplementary Note 3).
3. Our DFT calculation suggest a barrier-less step of C-H activation in CH_4 once the intermediate vanadium-based oxidant is electrochemically created (Supplementary Fig. 13a). This indicates an uncommonly fast kinetics of C-H activation, which is in accordance with a turnover-limiting *E* step.

In spite of the above-listed evidence, it is true that a homogenous electrocatalysis with a turnover-limiting *E* step is rare. However, this may not be impossible. Below we would like to offer our explanation why a turnover-limiting *E* step is plausible in our scenario.

Potential criticism 1: a rate-limiting *E* step is unlikely since the following C-H activation in CH_4 is difficult.

Homolytic cleavage of C-H bond in CH_4 is difficult in general. This demands a reactive and energetic intermediate oxidant, generated by electrochemistry. Due to the high

reactivity and instability of such an intermediate oxidant, the electrochemical generation of such an intermediate could be difficult and kinetically slow.

This could also be understood based on the Marcus theory:[21]

$$k_{ET} = \frac{2\pi}{\hbar}|H_{AB}|^2 \frac{1}{\sqrt{4\pi\lambda k_B T}} exp\left(-\frac{(\lambda + \Delta G^o)^2}{4\lambda k_B T}\right) \quad (16)$$

Here k_{ET} denotes the rate of electron transfer, λ the reorganization energy, ΔG^o the Gibbs free energy difference at standard conditions, $|H_{AB}|$ the electronic coupling term between the initial state, (**1** + FTO electrode), and the final state (**2** + e^- on FTO electrode). While λ could be small as noted by the reviewer (see point 2 below), the energetic intermediate should possess a large, positive value of ΔG^o, which leads to a small value of k_{ET} in addition to a small population of reactive V-based intermediate during electrolysis. Both the small value of k_{ET} and the small steady-state concentrations of V-based intermediate can yield a catalysis with a turnover-limiting *E* step.

Moreover, the uncertainty related to $|H_{AB}|$ may also incur a small value of k_{ET} and subsequently a turnover-limiting *E* step. The electrode based on tin oxide, such as fluorine-doped tin oxide (FTO) employed in our experiments, is known for its sluggish kinetics of charge transfer as compared to graphite or metal-based electrodes (Pt, Au, etc.). In the tight-binding model of solid-state physics, the Fermi level of FTO primarily resides on the 5s orbital of Sn[22], which is of lower density of state and not directly exposed at the FTO-electrolyte interface. This possibly contributes to a decreased value of $|H_{AB}|$ and subsequently a small value of k_{ET}. For example, the classic single-electron redox couple of ferrocenium/ferrocene (Fc^+/Fc) possesses a charge transfer rate as low as 9 sec^{-1} on indium tin oxide[23], or a characteristic time constant of about 0.1~10 sec on FTO surface[24].

Those kinetics are surprisingly slow for the typically presumed outer-sphere charge transfer of Fc^{+}/Fc. In our experiments, the observed TOF for vanadium catalyst reaches about 1,300 hr^{-1}, or about 0.4 sec^{-1} (Supplementary Table 1). Since our vanadium-based electrocatalyst can be understandably slower in kinetics than Fc^{+}/Fc couple, our observed TOF value is indeed commensurable, at least not impossible, with a catalysis of a turnover-limiting *E* step.

Last but not least, we want to note that our DFT calculation predicts a barrier-less C-H activation once the intermediate oxidant is electrochemically generated (Fig. 4e and Supplementary Fig. 13a), in contrast to the typical presumption that C-H activation in CH_4 must be kinetically slow. This point further suggests homogenous electrocatalysis with a turnover-limiting *E* step is possible.

Potential criticism 2: a catalysis based on a rate-limiting *E* step is unlikely because the *E* step is equivalent to a one-electron oxidation of sulfate ligand, which should possess low reorganization energy.

We in general agree that the one-electron oxidation of **1** should have small value of reorganization energy λ. Indeed, this is supported by the low apparent activation energy observed in our experiments (Fig. 3d). However, as the above discussion suggests, a small value of λ does not necessarily warrant a high value of k_{ET}. Additionally, even a small value of λ does hypothetically leads to a high value of k_{ET}, a catalysis with turnover-limiting *E* step is still possible, given the predicted barrier-less kinetics of C-H activation in CH_4 once the intermediate oxidant is electrochemically generated (Fig. 4e and Supplementary Fig. 13a). Therefore, a low reorganization energy cannot exclude the possible existence of a rate-limiting *E* step in our system.

Supplementary Note 3

In this note, we aim to provide a more detailed explanation of our operando XAS data and how it connects with our proposed mechanism.

Before we discuss the reported XAS data, we would like to briefly explain the source of signal for operando XAS experiment. Under the total-fluorescence-yield mode of our experimental setup[25,26], the operando XAS picks up any V K-edge signals not only on the FTO electrode but also in the solution proximate to the FTO electrode, because the incident X-ray penetrates through the FTO electrode into the electrolyte solution for $10^0 \sim 10^1$ μm near the electrode[26]. Therefore, while typically in recent literature operando XAS experiment is employed to detect signals of heterogenous electrocatalysts on the electrode's surface[25], operando XAS can indeed detect signals for homogenous electrocatalyst in the solution near the electrode's surface under electrolysis.

Now let's turn our attention to the reported operando XAS results (Supplementary Fig. 11 and 12). At first glance, it is counter-intuitive to observe a decrease of V oxidation state under higher electrochemical potential, given the large thermodynamic driving force to re-oxidize V(IV) into V(V). But we want to remind that such re-oxidation event can only happen if the vanadium species manage to physically reach the electrode and initiate a charge-transfer event. Therefore, the direct implication to explain such an observation is that the vanadium species undergoes homogenous redox reaction during electrolysis, because a heterogenous variant cannot yield a detectable decrease of vanadium oxidation state under such an anodic potential.

The observed results are indeed consistent with our proposed mechanism shown in Fig. 1d and Supplementary Fig. 14. In our proposed mechanism, after a single-electron oxidation

of $V^{V,V}$ dimer into $V^{V,V\,\bullet+}$ cation radical, two-electron C-H activation of CH_4 proceeds and subsequently yields a $V^{IV,V}$ dimer in the solution. Such a homogenous $V^{IV,V}$ dimer needs to diffuse and physically contact the electrode, before it gets re-oxidized into the starting $V^{V,V}$ dimer. Therefore, the observed decrease of V oxidation state can be interpreted as the detection of $V^{IV,V}$ dimer before it gets regenerated. At more anodic electrode potentials, the $V^{IV,V}$ dimer gains a larger population and hence the average V oxidation number decreases more. The preceding $V^{V,V\,\bullet+}$ cation radical is not considered to contribute much to the change of V oxidation number, because $V^{V,V\,\bullet+}$ cation radical, even long-lived enough to be detectable, should exhibit similar V oxidation number as the starting $V^{V,V}$ dimer based on our DFT calculation results (Fig. 4e).

This above argument is reasonable from the technical side based on a quick estimate. For homogeneous electrocatalysis, the reaction-diffusion layer scales with $(D/TOF)^{1/2}$, i.e. the approximate distance that a reactive species can travel before it fulfills a turnover. As we have determined $D \sim 10^{-7}$ cm^2 s^{-1} and TOF ~ 483 hr^{-1} = 0.13 sec^{-1} at 1-bar CH_4, the reaction-diffusion layer is roughly 8.8 μm under electrolysis. Such a length scale is commensurate with the penetration depth of the hard X-ray employed in current study[26]. Hence the proposed $V^{IV,V}$ dimer after C-H activation should be detectable in our operando XAS setup.

Putting all the pieces together, our operando XAS data cannot be explained by a process of heterogenous electrocatalysis and strongly suggest a homogeneous mechanism. The counter-intuitive XAS data indeed support our proposed mechanism by detecting the $V^{IV,V}$ dimer after the C-H activation of CH_4. While further mechanistic study is definitely needed (and we will!), our existing XAS data are consistent with our proposed mechanism.

Supplementary References

1 Madic, C., Begun, G. M., Hahn, R. L., Launay, J. P. & Thiessen, W. E. Dimerization of aquadioxovanadium(V) ion in concentrated perchloric and sulfuric-acid media. *Inorg. Chem.* **23**, 469-476, (1984).

2 Díaz-Urrutia, C. & Ott, T. Activation of methane to CH_3^+: a selective industrial route to methanesulfonic acid. *Science* **363**, 1326-1329, (2019).

3 Sadow, A. D. & Tilley, T. D. Homogeneous catalysis with methane. A strategy for the hydromethylation of olefins based on the nondegenerate exchange of alkyl groups and sigma-bond metathesis at scandium. *J. Am. Chem. Soc.* **125**, 7971-7977, (2003).

4 Meyer, D. *et al.* Palladium complexes with pyrimidine-functionalized N-heterocyclic carbene ligands: synthesis, structure and catalytic activity. *Organometallics* **28**, 2142-2149, (2009).

5 O'Reilly, M. E., Kim, R. S., Oh, S. & Surendranath, Y. Catalytic methane monofunctionalization by an electrogenerated high-valent Pd intermediate. *ACS Cent. Sci.* **3**, 1174-1179, (2017).

6 Kim, R. S. & Surendranath, Y. Electrochemical reoxidation enables continuous methane-to-methanol catalysis with aqueous Pt salts. *ACS Cent. Sci.* **5**, 1179-1186, (2019).

7 Chan, S. I. *et al.* Efficient oxidation of methane to methanol by dioxygen mediated by tricopper clusters. *Angew. Chem. Int. Ed.* **52**, 3731-3735, (2013).

8 Periana, R. A. *et al.* A mercury-catalyzed, high-yield system for the oxidation of methane to methanol. *Science* **259**, 340-343, (1993).

9 Periana, R. A., Mironov, O., Taube, D., Bhalla, G. & Jones, C. J. Catalytic, oxidative condensation of CH_4 to CH_3COOH in one step via CH activation. *Science* **301**, 814-818, (2003).

10 Jones, C. J. *et al.* Selective oxidation of methane to methanol catalyzed, with C-H activation, by homogeneous, cationic gold. *Angew. Chem. Int. Ed.* **43**, 4626-4629, (2004).

11 Smith, K. T. *et al.* Catalytic borylation of methane. *Science* **351**, 1424-1427, (2016).

12 Periana, R. A. *et al.* Platinum catalysts for the high-yield oxidation of methane to a methanol derivative. *Science* **280**, 560-564, (1998).

13 Hu, A. H., Guo, J. J., Pan, H. & Zuo, Z. W. Selective functionalization of methane, ethane, and higher alkanes by cerium photocatalysis. *Science* **361**, 668-672, (2018).

14 Zimmermann, T., Soorholtz, M., Bilke, M. & Schüth, F. Selective methane oxidation catalyzed by platinum salts in oleum at turnover frequencies of large-scale industrial processes. *J. Am. Chem. Soc.* **138**, 12395-12400, (2016).

15 Munz, D., Meyer, D. & Strassner, T. Methane CH activation by palladium complexes with chelating bis(NHC) ligands: a DFT study. *Organometallics* **32**, 3469-3480, (2013).

16 Fuller, J. T. *et al.* Catalytic mechanism and efficiency of methane oxidation by Hg(II) in sulfuric acid and comparison to radical initiated conditions. *ACS Catal.* **6**, 4312-4322, (2016).

17 Chempath, S. & Bell, A. T. Density functional theory analysis of the reaction pathway for methane oxidation to acetic acid catalyzed by Pd^{2+} in sulfuric acid. *J. Am. Chem. Soc.* **128**, 4650-4657, (2006).

18 Mironov, O. A. *et al.* Using reduced catalysts for oxidation reactions: mechanistic studies of the "Periana-Catalytica" system for CH_4 oxidation. *J. Am. Chem. Soc.* **135**, 14644-14658, (2013).
19 Costentin, C., Drouet, S., Robert, M. & Saveant, J. M. Turnover numbers, turnover frequencies, and overpotential in molecular catalysis of electrochemical reactions. Cyclic voltammetry and preparative-scale electrolysis. *J. Am. Chem. Soc.* **134**, 11235-11242, (2012).
20 Savéant, J. M. *Elements of molecular and biomolecular electrochemistry. An electrochemical approach to electron transfer chemistry*. (Wiley, New Jersey, 2006).
21 Bolton, J. R. & Archer, M. D. *Electron transfer in inorganic, organic, and biological systems. Chapter 2, pp 7-23. Basic electron-transfer theory*. (The American Chemical Society, 1991).
22 Robertson, J. Electronic structure of SnO_2, GeO_2, PbO_2, TeO_2 and MgF_2. *J. Phys. C: Solid State Phy.* **12**, 4767-4776, (1979).
23 Hanna, C. M., Sanborn, C. D., Ardo, S. & Yang, J. Y. Interfacial electron transfer of ferrocene immobilized onto indium tin oxide through covalent and noncovalent interactions. *Acs Appl. Mater. Interfaces* **10**, 13211-13217, (2018).
24 Hamann, T. W., Farha, O. K. & Hupp, J. T. Outer-sphere redox couples as shuttles in dye-sensitized solar cells. Performance enhancement based on photoelectrode modification via atomic layer deposition. *J. Phys. Chem. C* **112**, 19756-19764, (2008).
25 Bai, L. C., Hsu, C. S., Alexander, D. T. L., Chen, H. M. & Hu, X. L. A cobalt-iron double-atom catalyst for the oxygen evolution reaction. *J. Am. Chem. Soc.* **141**, 14190-14199, (2019).
26 Lin, F. *et al.* Synchrotron X-ray analytical techniques for atudying materials electrochemistry in rechargeable batteries. *Chem. Rev.* **117**, 13123-13186, (2017).
